# Asymmetric Transcript Discovery by RNA-seq in *C*. *elegans* Blastomeres Identifies *neg-1*, a Gene Important for Anterior Morphogenesis

**DOI:** 10.1371/journal.pgen.1005117

**Published:** 2015-04-13

**Authors:** Erin Osborne Nishimura, Jay C. Zhang, Adam D. Werts, Bob Goldstein, Jason D. Lieb

**Affiliations:** 1 Lineberger Comprehensive Cancer Center, University of North Carolina, Chapel Hill, Chapel Hill, North Carolina, United States of America; 2 Department of Biology, University of North Carolina, Chapel Hill, Chapel Hill, North Carolina, United States of America; 3 Department of Human Genetics, University of Chicago, Chicago, Illinois, United States of America; Harvard University, UNITED STATES

## Abstract

After fertilization but prior to the onset of zygotic transcription, the *C*. *elegans* zygote cleaves asymmetrically to create the anterior AB and posterior P_1_ blastomeres, each of which goes on to generate distinct cell lineages. To understand how patterns of RNA inheritance and abundance arise after this first asymmetric cell division, we pooled hand-dissected AB and P_1_ blastomeres and performed RNA-seq. Our approach identified over 200 asymmetrically abundant mRNA transcripts. We confirmed symmetric or asymmetric abundance patterns for a subset of these transcripts using smFISH. smFISH also revealed heterogeneous subcellular patterning of the P_1_-enriched transcripts *chs-1* and *bpl-1*. We screened transcripts enriched in a given blastomere for embryonic defects using RNAi. The gene *neg-1 (F32D1*.*6)* encoded an AB-enriched (anterior) transcript and was required for proper morphology of anterior tissues. In addition, analysis of the asymmetric transcripts yielded clues regarding the post-transcriptional mechanisms that control cellular mRNA abundance during asymmetric cell divisions, which are common in developing organisms.

## Author Summary

At key moments in development, asymmetric cell divisions give rise to daughter cells of differing characteristics, a process that promotes cell-type diversity in complex organisms. The first cell division of the *C*. *elegans* early embryo is a powerful model for understanding asymmetric cell division because the timing of divisions and the placement of their division planes are precise and reproducible. We surveyed the mRNA content of each daughter cell in the *C*. *elegans* 2-cell embryo using low-input RNA sequencing. We identified several hundred asymmetric transcripts and tested them for functions in development. We found that the gene *neg-1* produced mRNA and protein preferentially on the anterior (head-side) of 2-cell and 4-cell stage embryos and that loss of *neg-1* led to consequences in anterior morphogenesis later in development. We also analyzed the asymmetric transcripts using quantitative microscopy, bioinformatics comparisons with previously existing datasets, and RNA sequence motif discovery to gain insight to the mechanisms by which asymmetric abundance patterns arise.

## Introduction

**Fig 1:**
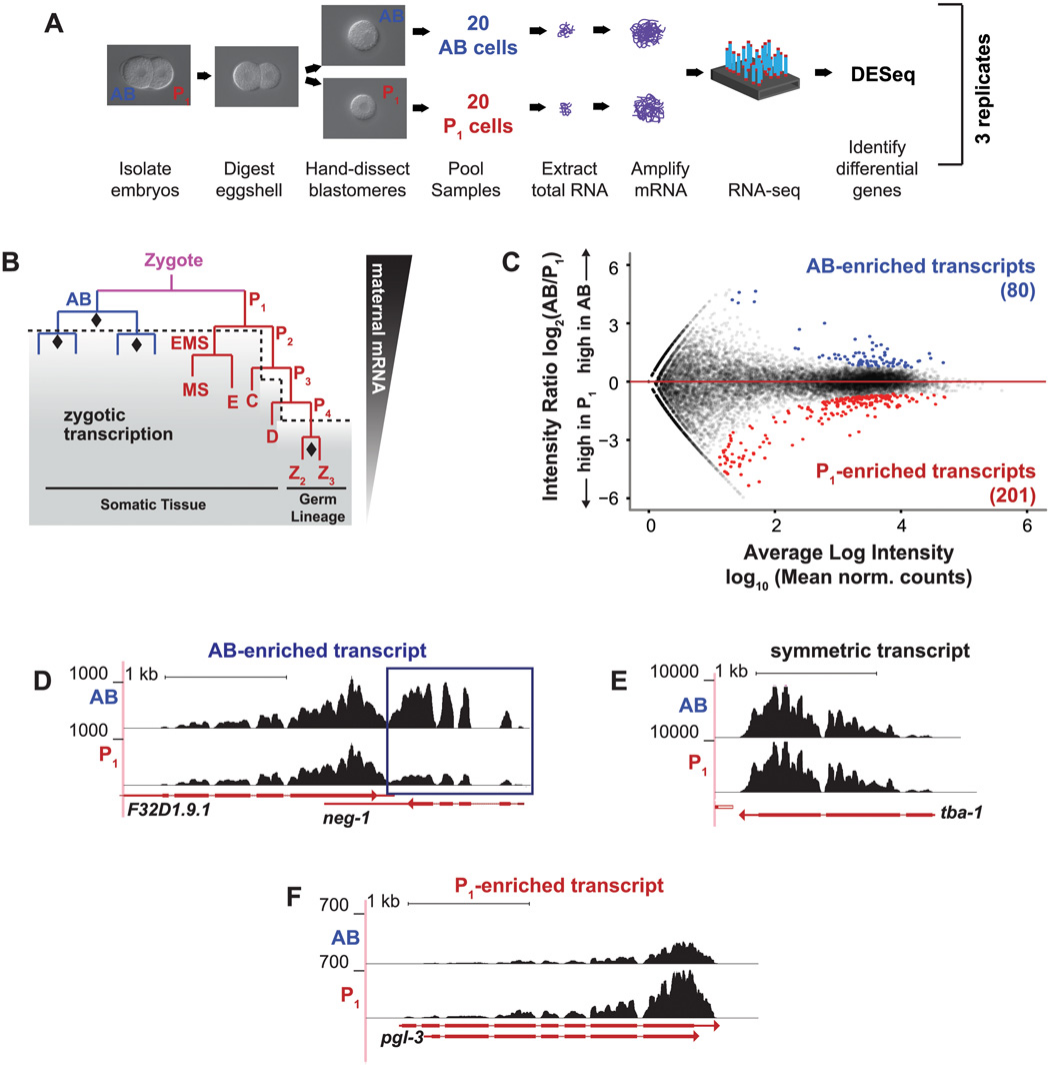
Differential transcript abundance in AB and P_1_ blastomeres following the first embryonic division. **(A)** *C*. *elegans* wild type embryos were harvested from gravid adults. AB and P_1_ blastomeres were separated by microdissection, and mRNA was extracted, amplified and sequenced. **(B)** The first cell division is asymmetric and creates the anterior AB cell and posterior P_1_ cell. RNA in the AB and P_1_ cells is exclusively maternal prior to the onset of zygotic transcription (dashed line, shading). Symmetric cell divisions are marked by diamonds. **(C)** For each gene, the normalized ratio of RNA-seq read counts between P_1_ and AB is plotted against the mean normalized read count for each transcript (Materials and Methods). Asymmetrically abundant transcripts are shown in blue (AB-enriched) and red (P_1_-enriched). **(D)** RNA-amp-seq traces for an AB-enriched transcript (*neg-1*, boxed in blue) **(E)** Same as **D** for a symmetric transcript (*tba-1)* **(F)** Same as **D** for a P_1_-enriched transcript (*pgl-3)*.

Asymmetric cell divisions produce daughter cells of different size, molecular content, or developmental potential. These events promote tissue-type diversity in developing embryos, specify terminal differentiation, and allow for the maintenance of adult tissues [1, 2]. Asymmetric cell divisions trigger divergent cell fates through the unequal distribution of cell fate determinants or by moving daughter cells into different morphogen fields [3, 4]. Searches to identify intrinsic cell fate determinants and the mechanisms that guide their asymmetric distribution have been difficult to adapt to high-throughput strategies. A key challenge is separating daughter cells with sufficient purity and yield for genome-wide and proteome-wide assays. We sought to overcome this challenge in *Caenorhabditis elegans* by coupling a low-input RNA-seq protocol [5, 6] with hand-dissection of blastomeres, which ensures absolute purity in each pool of isolated cells (Fig 1A).

In *C*. *elegans*, an asymmetric cell division cleaves the recently-fertilized zygote into a larger anterior AB cell and a smaller posterior P_1_ cell, each of which has distinctive characteristics and fates (Fig 1B) [7]. The AB cell undergoes a series of rapid, symmetric cell divisions to eventually produce hypodermal, muscle, and neuronal tissue [8–10]. The smaller daughter cell, P_1_, is rich in perinuclear bodies of ribonucleoparticles called P granules. Unlike the AB cell that undergoes symmetric cell division, the P_1_ cell initiates a series of successive asymmetric cell divisions in which the smaller cell progressively inherits P granules and primordial germ cell fate and the larger cell becomes somatic tissues such as hypodermal, intestine, neuronal, and muscle tissues [8, 11, 12]. The somatic branches of the AB- and P_1_-lineages are transcriptionally active but the germ cell branch of the P_1_ lineage remains largely transcriptionally quiescent with some notable exceptions [13–16] (Fig 1B).

Four features make the first division of *C*. *elegans* embryogenesis an excellent model for studying the apportionment of mRNA through asymmetric cell division. First, the cell divisions of the early embryo are invariant and cell fates are precisely mapped [8, 13, 15–19], allowing one to connect any mRNA asymmetries to functional consequences later in embryogenesis. Second, many of the mechanisms and proteins responsible for polarity and asymmetry have been identified, ultimately allowing the mechanisms that drive mRNA partitioning to be placed into an established framework [20]. Third, zygotic transcription does not initiate until the 4-cell stage and becomes widespread at the 16-cell stage [13, 15, 21], meaning that RNA segregation, stabilization, and degradation can be observed independent of *de novo* transcription. Fourth, a few transcripts have been previously identified as asymmetrically abundant at this stage using *in situ* hybridization (*mex-3*, *pos-1*) allowing for verification by independent methods [22–24].

A previous study [16] identified 14 AB-enriched mRNA transcripts and 4 P_1_-enriched transcripts using a single-cell RNA-seq approach, in which only one cell is measured in each sample. Because our goal was to obtain as comprehensive a set of asymmetrically patterned genes as possible and to guard against cell-to-cell variability and noise, each of our samples consisted of a pool of 20 individual hand-dissected cells. By pooling AB and P_1_ cells, we were able to achieve lower variance in our samples (S2 Fig), allowing us to identify 80 AB-enriched and 201 P_1_ enriched transcripts. This is consistent with other studies showing that pooling cells to quantities of 20-cells or more buffers against cell-to-cell variation and yields more reliable and reproducible quantification [25]. The larger number of asymmetric transcripts we discovered allowed us to derive common properties of mRNAs that are asymmetrically partitioned in the early embryo. We complemented our transcriptome profiling approach with quantitative microscopy to verify our findings and further resolve patterns of mRNA distribution. We also used our dataset to identify *neg-1* as a gene newly discovered to be important for anterior morphology.

## Results

### Determination of RNA abundance in AB and P_1_ blastomeres by RNA-amp-seq

We developed a low input RNA-seq protocol (RNA-amp-seq) that maintains relative transcript abundance and minimizes the potential for contamination. Total RNA was amplified by *in vitro* transcription (Eberwine amplification) of a low input cDNA pool [26], and amplified RNA was subjected to RNA-seq. Transcript abundance determined by RNA-amp-seq had a high correlation to standard RNA-seq (r^2^ = 0.84; S1A Fig) and preserved calls of enriched and depleted transcripts (S1B Fig). Although there is high concordance between amplified and unamplified samples, some transcripts may not be captured by the amplification procedure, resulting in false negatives. These results agree with previous studies demonstrating that *in vitro* transcription amplifies whole transcriptomes linearly, preserving relative transcript abundances [6, 16, 27].

To obtain AB and P_1_ specific transcriptomes, we isolated AB and P_1_ from 2-cell stage embryos. Two-cell stage embryos were removed from their eggshells with chitinase and chymotrypsin treatment and were extracted from the remaining envelope through mechanical sheering. AB and P_1_ cells were separated using a hollow-tipped glass needle attached to a mouth aspirator [28, 29]. Once separated, the two blastomeres were clearly distinguishable by their relative sizes. Twenty matched AB and P_1_ cells were pooled for each of three replicates (Fig 1A). We performed RNA-amp-seq and identified transcripts with statistically significant differential abundances [30–33]. Eighty of these RNAs were enriched in the anterior AB cell and 201 in the posterior P_1_ cell (Fig 1C–1G) with a Benjamani-Hochberg-adjusted *P* value of less than 0.10. For some analyses, these were ranked by adjusted *P* value (S1 Dataset, S1 Table).

### Complementary techniques confirm asymmetrically abundant transcripts

We used several independent methods to confirm subsets of our identified asymmetrically abundant mRNA transcripts.

**Fig 2:**
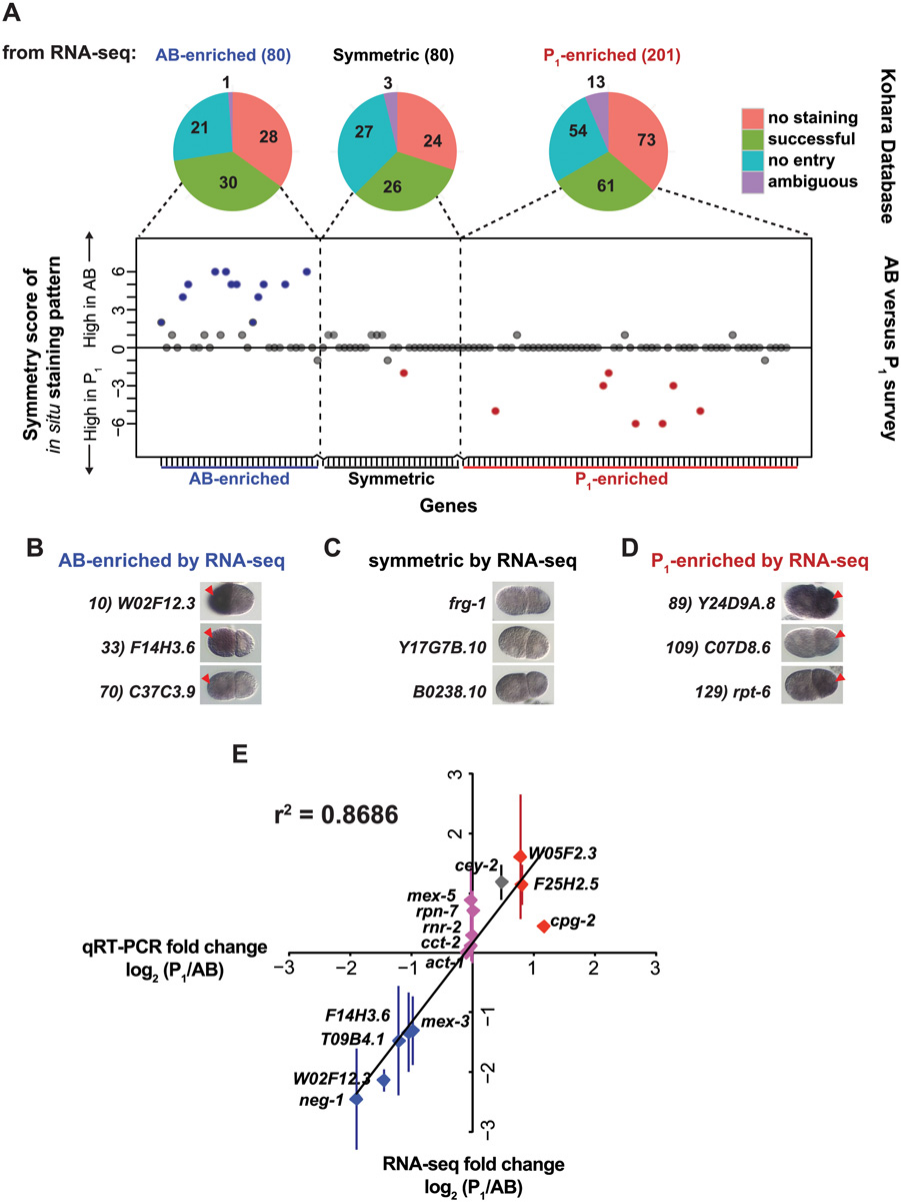
Complementary methods, *in situ* hybridization and qRT-PCR, support RNA-seq measurements. **(A)** The 80 AB-enriched, the 201 P_1_-enriched, and a subset of the middle 80 symmetric transcripts (S3 Dataset) were queried in a publically available collection of *in situ* hybridization images [34]. Pie charts show the proportion of transcripts with no entry (blue), successful staining (green), those that were in the database but showed no signal in any of the samples (No staining, red), and those that were otherwise interpretable due to overexposure, focus, or other problems (ambiguous, purple). Successfully stained entries were scored in a blind survey, and their results are plotted for each entry queried. Transcripts with scores higher than 2 are marked in blue and lower than 2 are marked in red. **(B)** Representative *in situ* hybridization images of AB-enriched transcripts [34]. The anterior AB cell is always oriented to the left of the posterior P_1_ cell. Red arrowheads indicate the cell with higher observed signal. All images in B, C, and D were taken from the Nematode Expression Data Base (http://nematode.lab.nig.ac.jp/db2/index.php). **(C)** As in **B** for symmetric transcripts **(D)** As in **B** for P_1_-enriched transcripts. **(E)** qRT-PCR was performed on pools of 5 AB and matched P_1_ cells for a subset of genes classified as AB-enriched (purple), P_1_-enriched (red), and symmetric (pink) by RNA-seq. Fold-change (P_1_/AB) as measured by qRT-PCR (Y-axis) and by RNA-seq (X-axis) is plotted. Each data point represents at least 2 independent biological samples, each of which was measured in at least 2 technical replicates; standard error of the mean is shown for biological replicates.

First, we compared asymmetric transcripts to an existing database of *C*. *elegans* RNA *in situ* hybridization images [34]. Note that the *in situ* database was created systematically and was not designed or optimized to detect transcript asymmetries at the two-cell stage. Therefore, a lack of a discernable positive signal was not evidence of a false positive in our dataset. We queried *in situ* hybridization entries for our 80 AB-enriched, 201 P_1_-enriched, and the 80 symmetric transcripts with the most uniform distribution (of 7664) (Fig 2A–2D, S2 Dataset). Many transcripts were absent from the online database, showed no staining, or were uninterpretable. Entries that had successful staining were scored in a blind survey that was used to generate a symmetry score. We found a strong association between entries that were identified as AB-enriched or P_1_-enriched by our RNA-seq analysis and those that yielded either high or low symmetry scores, respectively (Fig 2A–2D, S2 Dataset). Rates among all *in situ* images with positive staining were less than 3% AB-enriched and less than 1% P_1_-enriched (97% symmetric) when we queried a random set of 100 genes present at the 2-cell stage. Given that the hybridizations were not optimized for detecting cell-to-cell variation at the 2-cell stage of development, it is remarkable that so many of our transcripts were validated by this resource.

Second, we performed qRT-PCR on pools of five blastomeres to measure the abundance of individual RNA transcripts. We selected transcripts to test from each set of genes: AB-enriched, P_1_-enriched, and symmetric. The ratio of transcript abundance (P_1_/AB) fold change determined by qRT-PCR was highly correlated with that determined by RNA-seq (r^2^ = 0.86) (Fig 2D). One caveat to this technique is that reliable quantification requires a linear standard dilution series for each primer set. Only transcripts with mean RNA-seq abundance values over 15,000 passed this requirement and were quantifiable by this method.

We also compared our data to the few transcripts whose mRNA localization had been previously characterized at the 2-cell stage of development. The *mex-3* mRNA transcript is preferentially abundant in the AB cell [23], *pos-1* is P_1_-enriched [24], and *cey-2* appears initially uniform in early 2-cell stage but becomes P_1_-enriched in late 2-cell stage [22]. Of these transcripts, *mex-3* appeared in our set of statistically significant AB-enriched transcripts. However, neither *cey-2* nor *pos-1* appeared on our list of P_1_-enriched transcripts. Though *cey-2* and *pos-1* were enriched 1.4 fold and 1.2 fold in the P_1_ cell in our dataset, they did not meet the conservative FDR-adjusted *P* value threshold we used. The transcripts for *ama-1*, *eft-4*, *dpy-3*, *act-1*, *and tba-1* were previously characterized as having uniform distribution at the 2-cell stage [22] and appear among our symmetrically patterned transcripts. Further, a recent study reported that a transcript we identified as AB-enriched, *W02F12*.*3*, was quantifiable as AB-enriched by microscopy [35].

There was high concordance between the AB and P_1_-enriched transcripts reported in Hashimshony et al. [16] and those identified by our study (S2B and S2C, Fig). Five of the 14 genes previously identified as AB-enriched were also classified as AB-enriched in our study, and 3 of 4 P_1_-enriched genes previously identified were also in our set of 201 P_1_-enriched genes (S2B and S2C, Fig). Both Hashimshony et al. and our study failed to identify *cey-2* and *pos-1* transcripts as P_1_-enriched, which had been previously shown by *in situ* hybridization [22, 24].

### Single-molecule RNA FISH revealed subcellular localization of asymmetric transcripts

**Fig 3:**
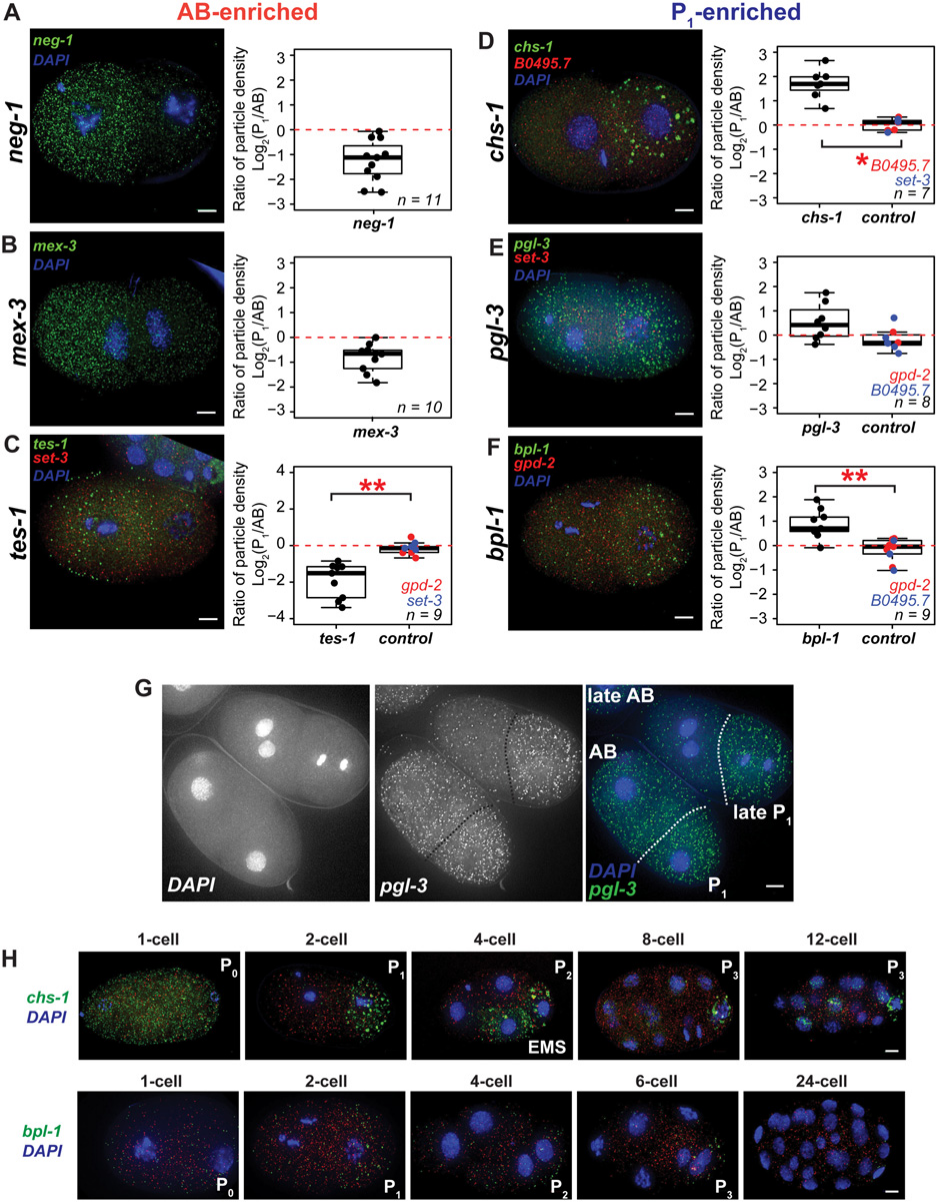
Quantitative *in situ* hybridization using smFISH probes. **(A-F)** Three AB-enriched, three P_1_-enriched, and three symmetric transcripts were imaged using sets of smFISH probes. **(A-B)** Images of wild type (*N2)* embryos grown at 20°C, fixed, and hybridized with *mex-3* or *neg-1* probes are shown. Ratios of particle density are reported (see Materials and Methods). Positive values indicate greater P_1_ cell particle densities; negative values indicate greater AB cell densities. *neg-1* ranked #4 and *mex-3* ranked #42 of 80 AB-enriched transcripts when ordered by *P* value. *neg-1* (n = 11); *mex-3* (n = 10). **(C-F)** *tes-1*, *chs-1*, *pgl-3*, and *bpl-1* were multiplexed with control probe sets labeled with a different fluorophore (hybridizing to either *gpd-2*, *set-3*, or *B0495*.*7)* to image both an asymmetric and symmetric transcript within the same embryo. *tes-*1 was ranked #77 of 80 AB-enriched transcripts. *chs-1* (rank #1), *pgl-3* (rank #32), and *bpl1* (rank #170) were among 201 P_1_-enriched transcripts. *P < 0.05; **P < 0.005, calculated using the Wilcoxon signed-rank test for paired samples. *tes-1* (n = 9); *chs-1* (n = 8); *pgl-3* (n = 7); *bpl-1* (n = 9). **(G)** P_1_-biased density of *pgl-3* particles was more dramatic in late 2-cell stage embryos undergoing mitosis. **(H)** *chs-1* and *bpl-1* transcripts in fixed embryos over several stages of early embryonic development. All embryos are shown counterstained with DAPI. Maximal projections of optical sections are shown. Scale bars 5 μm.

The limitations of qRT-PCR and the publicly available *in situ* hybridization datasets motivated us to employ an alternative method of measuring mRNA transcript abundance. We performed quantitative *in situ* hybridization, also known as single-molecule FISH (smFISH). In separate experiments, we used probes designed to hybridize to three AB-enriched transcripts (*mex-3*, rank #4; *neg-1*, rank #42; *tes-1*, rank #77 of 80 total), three P_1_-enriched transcripts (*chs-1*, rank #1;* pgl-3*, rank #32; *bpl-1*, rank #170 of 201 total), and three symmetric transcripts (*gpd-2*, *set-3*, and *B0495*.*7*). We chose *mex-3* because it has been previously reported to be AB-enriched and *neg-1* because of our interest in this gene specifically. Other candidates were chosen to span a range of expression levels and to represent asymmetric transcripts at the top and bottom of our RNA-seq-based *P* value ranked lists. For each probe set, in each cell of the 2-cell embryo, both the number of fluorescent particles and the total fluorescence generated by the particles were quantified (Fig 3A–3F, S4A and S4B Fig). We report the ratio of “particle density”, where “particle density” is the volume-normalized count of fluorescent particles in the P_1_ cell as compared to the AB cell (Fig 3A–3F). In the case of *mex-3*, which is known to be enriched in AB, and *neg-1*, which was discovered to be AB-enriched by RNA-seq in this study, quantitative analysis confirmed AB enrichment (Fig 3A and 3B).

We next performed multiplex FISH with dual probe sets, which allowed us to compare an asymmetric transcript to a symmetric transcript within the same embryo. This approach provided an internal control and buffered against variations in hybridization efficiency. We tested for significant differences in the ratios of particle densities (P_1_/AB) of *tes-1* (AB-enriched) and *chs-1*, *pgl-3*, and *bpl-1* (P_1_-enriched) relative to *gpd-2*, *set-3*, or *B0495*.*7* (symmetric transcripts). The three symmetric transcripts were nearly always within a 2-fold range of abundance in the AB and P_1_ cells (Fig 3C–3F). In contrast, *tes-1* was up to 8-fold enriched in the AB cell relative to P_1_ (Fig 3C), confirming AB enrichment. This is important because *tes-1* is a low-abundance transcript that is not reliably detectable by qRT-PCR. It is the 77th of 80 AB-enriched asymmetric transcripts ranked by *P* value. Thus, we have observed that even transcripts near the bottom of our asymmetrically abundant lists can exhibit reproducible, quantitative, and microscopically verifiable asymmetric patterns.

Three transcripts that were P_1_-enriched by RNA-seq were tested by smFISH (*chs-1*, *pgl-3*, and *bpl-1)*. Of these, *chs-1* and *bpl-1* transcripts were P_1_-biased relative to internal controls (Fig 3D–3F). *chs-1* and *bpl-1* are ranked at the top and the bottom of the RNA-seq P_1_-enriched list of transcripts respectively. *pgl-3* was also tested but did not show statistically significant differences between an internal control (Fig 3E). Instead, *pgl-3* showed high variability in P_1_-enrichment ranging from marginal AB-enrichment to 4-fold higher P_1_–enriched particle density. We noticed that cells with higher ratios of P_1_ particle density were often later 2-cell stage embryos (Fig 3H) indicating that variability may be due to a greater degree of asymmetry in older 2-cell embryos, and a lesser degree in 1-cell embryos.

We also noted a qualitative difference in the *chs-1* hybridization signal. The fluorescence particles appeared much larger than signal produced from other transcripts. This granular pattern was suggestive of P granule localization (Fig 3D–3I). Because of their granular nature, we were not able to quantitate these transcripts with reliable single molecule resolution. For this reason we also measured total fluorescence for all our experiments. However, even in the case of *chs-1*, which had the most notable granular patterning, the ratios and statistics calculated from total fluorescence density measurements closely paralleled measurements for particle density (S4 Fig).

### *chs-1*, *pgl-3*, and, *bpl-1* continue to associate with posterior cells in subsequent embryonic cell divisions

smFISH allowed us to observe RNA abundance patterns in embryos throughout development. The *chs-1*, *pgl-1*, and *bpl-1* transcripts exhibited uniform transcript abundance in 1-cell stage embryos whereas transcripts in 2-cell stage embryos were localized asymmetrically (Fig 3G and 3H, S5 Fig). This suggests that differential transcript degradation or stabilization may contribute to pattern these particular mRNAs. Cell-specific distributions continued for *chs-1* and *bpl-1* transcripts, with particles concentrated in one or two posterior cells over the course of several cell divisions (Fig 3H).

### Time-lapse microscopy following dsRNA treatment reveals a function for neg-1 in development

**Fig 4:**
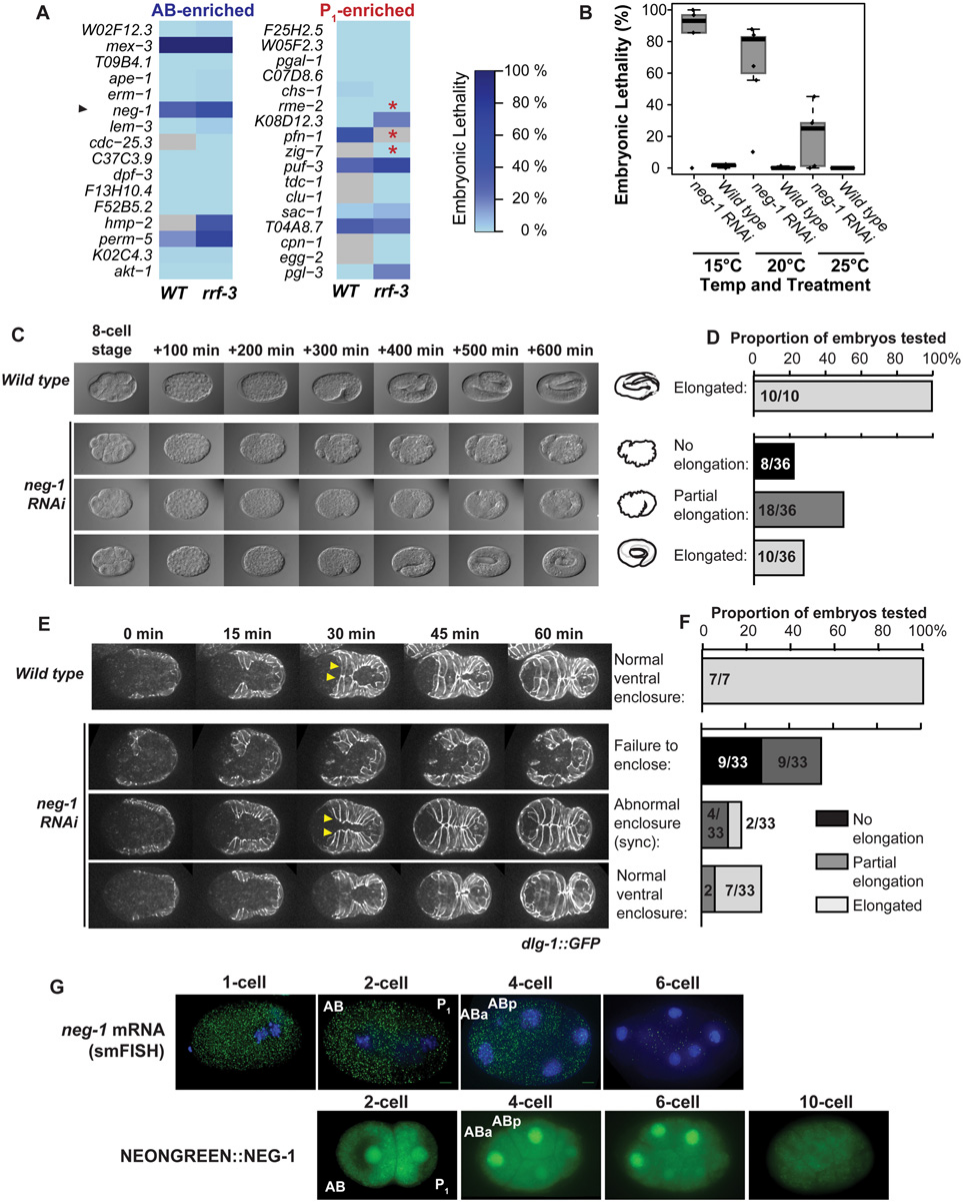
*neg-1* is required for morphogenesis. **(A)** The embryonic lethality resulting from RNAi depletion of asymmetrically abundant transcripts in wild type *(N2)* and RNAi sensitive *(rrf-3)* strains is shown. Embryonic lethality is depicted using the illustrated colorscale. Samples not tested are colored in gray. Worms that showed reduced brood size in response to RNAi feeding are marked with a red asterisk. **(B)** *neg-1* dsRNA injection is lethal and cold sensitive. Worms were grown at 20°C, injected with dsRNA or left uninjected at L4, and then moved to 15°C, 20°C, or 25°C. Embryonic lethality for the resulting embryos was scored for 3–4 replicates of 6 worms each. **(C)** Time-lapse microscopy of embryonic development from *neg-1* dsRNA-injected mothers at 20°C. Wild type embryos hatched normally under these conditions, but *neg-1* depleted embryos exhibited a range of elongation phenotypes from showing no signs of elongation, to partial elongation, to complete elongation. **(D)** Tabulation of embryo developmental phenotypes shown in (C). **(E)** *dlg-1*::*GFP* embryos depleted of *neg-1* were imaged and compared by time lapse microscopy at 20°C to wild type embryos. *dlg-1*::*GFP* is a fluorescent marker for apical adherens junctions of the hypodermal and gut epithelial cells. *neg-1* depletion disrupted ventral enclosure. Leader cells are noted by yellow arrowheads. **(F)** Tabulation of the *dlg-1*::*GFP* phenotypes shown in (E). Hypodermal cell defects were predictive of a failure to hatch. (G) Visualization of *neg-1* mRNA transcripts in fixed, wild type (*N2*) embryos using smFISH probes hybridized to *neg-1* and DAPI as a nuclear stain. Scale bar 5 μm. Below, live embryos expressing NEONGREEN::NEG-1 driven from a construct that included the full length NEG-1 3’UTR and 100 bp of downstream sequence.

We hypothesized that genes encoding asymmetrically abundant transcripts might have lineage-specific roles in the development of the early embryo. At least one known example supports this idea. MEX-3 mRNA and protein are both distributed preferentially to the AB cell and *mex-3* is required for proper AB lineage specification. We used RNAi to survey the knockdown phenotypes of 33 of our asymmetrically abundant transcripts (Fig 4A). Though RNAi assessments have been performed in the early embryo for some of these genes as part of systematic studies [36–42], we re-tested these 33 genes and scored and ranked relative embryonic lethality under uniform and controlled conditions. RNAi corresponding to 9 of the 33 genes tested yielded greater than 5% embryonic lethality in either wild type (*N2*) or sensitized (*rrf-3)* worms [40] (Fig 4A).

We next tested whether any of these 9 genes with embryonic lethality phenotypes were involved in lineage-specific functions by performing timecourse microscopy after dsRNA injection. RNAi to *neg-1* yielded the most dramatic anatomical defects, so we selected this gene for further study (Fig 4B–4D). *neg-1* encodes a small protein of unknown function predicted to contain an unstructured N-terminus and a potentially structured and positively charged C-terminus [43]. The only known homologs to this protein occur in *C*. *elegans* and *C*. *brenneri*. *F32D1*.*6* has recently been named *neg-1* (*Negative Effect on Gut development 1)*.

### *neg-1* is required for morphogenesis

We found that *neg-1* RNA was enriched and highly abundant in the anterior AB cell (Fig 1D, Fig 2E, Fig 3A). Disruption of *neg-1* by RNAi led to partially-penetrant embryonic lethality, with a median lethality rate of 96% by injection at 15°C, 81% at 20°C and 30% at 25°C (Fig 4B), and thus was cold-sensitive. Lethality was lower by RNAi feeding (30% at 20°C), which is consistent with previously published studies of *neg-1* RNAi feeding, which reported 40% lethality [44] (Fig 4A).

To achieve a more detailed characterization of the cause of embryonic lethality, we performed time-lapse microscopy on embryos from mothers that either were or were not injected with *neg-1* dsRNA (Materials and Methods) (Fig 4C and 4D). These experiments were conducted at 20°C. Among *neg-1* depleted embryos, 22.2% arrested without apparent signs of elongation, 50% arrested with partial elongation and 27.7% elongated. In embryos undergoing partial elongation, we noticed a failure of the cells in the anterior of the embryo to enclose, which likely caused the elongation defect. During this time, development of the posterior continued normally (Fig 4C and 4D). This is reminiscent of *hammerhead* (*hmr-1)* mutation in that the hypodermis failed to enclose the anteroventral regions of the embryo leading to a failure of elongation [45]. It is also similar to *humpback* (*hmp-1*, *hmp-2)* phenotypes although we did not observe the classic dorsal rippling or bulging that is typical of those mutants [8, 45, 46].

We suspected that the primary defect in *neg-1* depleted embryos was a failure of the hypodermis to fully enclose. During elongation, hypodermal cells encircle the embryo by extending from the dorsal half of the embryo and expanding to the ventral side, meeting and forming junctions at the ventral midline [47]. Once the ventral enclosure is complete, circumferential constriction of seam cells (a file of mid-body cells) and hypodermal cells powers the extension of the body into an elongated shape [48]. To more precisely monitor hypodermal cell behavior in *neg-1* knock-downs, we performed time-lapse microscopy in a *dlg-1*::*GFP* strain that marks apical adherens junctions of epithelial cells of the hypodermis and gut [49]. We found that in 55% of *neg-1* RNAi-treated embryos (18/33), hypodermal cells were misshapen prior to ventral enclosure (Fig 4E and 4F). Within this group, 9 embryos failed to complete ventral enclosure and did not undergo elongation (Fig 4E and 4F). The other 9 did not complete ventral enclosure on the anterior end and only partially underwent elongation. In these embryos, elongation led to an organized posterior region of the worm but forced internal cells out through the unenclosed anterior leading to the hammerhead-like phenotype. Interestingly, 18% (6/33) of embryos exhibited hypodermal cell enclosure that was largely normal, but mistimed. Typically, two actin-rich “leader” cells on flanking sides of the body are the first to meet at the ventral midline and are then followed by the more-posterior hypodermal cells. In the subset of embryos with defective timing, the leader cells and posterior cells met synchronously at the ventral midline. Of these 6 mistimed-enclosure embryos, 4 failed to complete elongation, whereas the other two hatched. The remainder of embryos (9/33, 27%) exhibited normal hypodermal morphogenesis, and of these, 7 of 9 hatched (Fig 4E and 4F). While there was a strong relation between abnormal behavior of hypodermal cells in *neg-1* compromised embryos and the likelihood of successful hatching (Fig 4F), the correlation was not perfect, suggesting that processes other than hypodermal cell behavior are also defective when *neg-1* is lacking.

mRNA abundance usually, but not always, correlates with protein production. To test whether anterior *neg-1* mRNA enrichment correlated with NEG-1 protein enrichment, we compared smFISH staining for *neg-1* mRNA to fluorescence in worms harboring a *NeonGreen*::*neg-1* protein fusion. As visualized by smFISH, *neg-1* mRNA appeared uniform in the 1-cell fertilized embryo, became localized to the anterior in the AB cell and its daughters, and then declined dramatically in signal during the 6–10-cell stages (Fig 4G). We observed NeonGreen fluorescence in the ABa and ABp nuclei at the 4-cell stage. NeonGreen persisted in the anterior nuclei of the 6-cell and 8-cell stage embryos, but then decreased by the 10–32-cell stages. Therefore, anterior enrichment of *neg-1* mRNA preceded fluorescent protein accumulation by one cell cycle, and the pattern of anterior-localization between mRNA and protein was correlated.

### Gene categories associated with AB-enriched transcripts are different from those associated with P_1_-enriched transcripts

**Fig 5:**
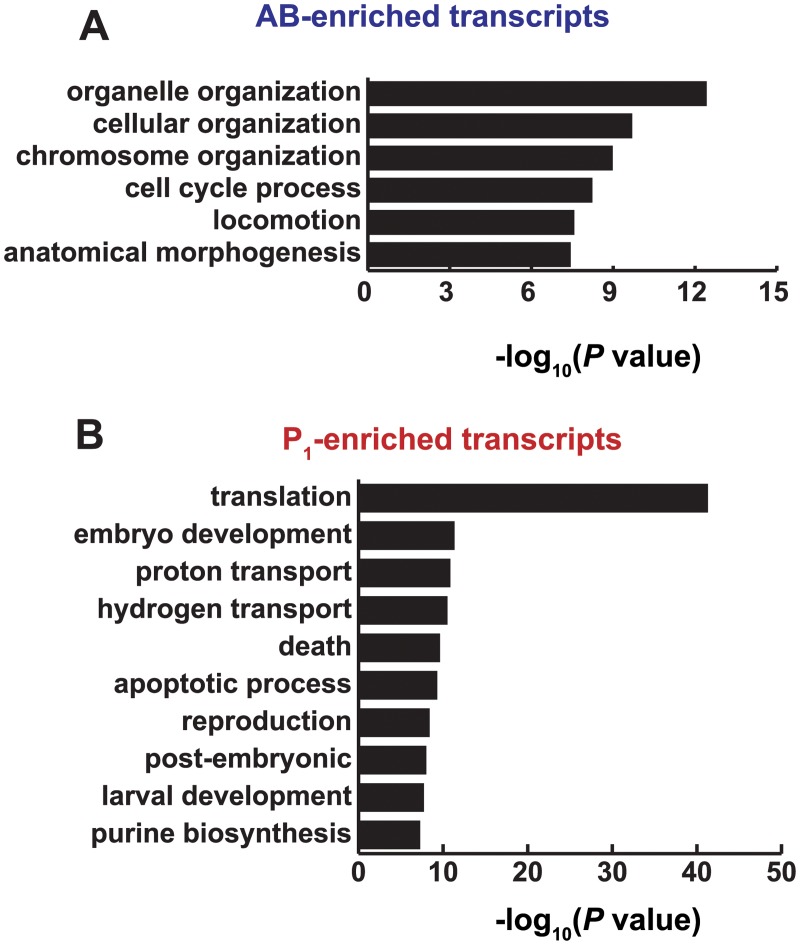
Functions associated with AB and P_1_-enriched transcripts. **(A)** GO ontology terms over-represented in genes whose transcripts were AB-enriched at the 2-cell stage of development. Categories were identified using the GOrilla algorithm for identifying categorical significance from ranked lists [50] and were summarized using the REViGO algorithm [51]. **(B)** Same as A for P_1_-enriched.

Having identified an anterior transcript required for embryogenesis and required for full function in many AB-derived hypodermal cells, we asked whether asymmetric transcripts had functions associated with the lineage in which they were enriched. Transcripts that accumulated in AB were enriched in GO biological process categories [50] that were distinct from the categories enriched in P_1_ transcripts (Fig 5A). Categories associated with the AB transcripts included organelle organization, cellular organization, chromosome organization, and cell-cycle processes, locomotion, and morphogenesis (Fig 5A, S5 Dataset). The AB cell lineage undergoes more rapid cell cycle progression than the P_1_ lineage [8,9,49,50], which explains the enrichment for genes involved in cell cycle processes.

Transcripts preferentially abundant in the posterior P_1_ cell were associated with translational control, embryo development, proton transport, and cell death (Fig 5A, S5 Dataset). Although translational control is prevalent throughout *C*. *elegans* embryogenesis, germ line development is especially dependent on translational control due to the transcriptional quiescence of this lineage until later stages of embryogenesis [12, 52, 53]. Competition and cooperation among RNA-binding proteins, some of which are asymmetrically distributed in the AB and P_1_ lineages, control translational repression and activation of maternally inherited mRNAs [54]. For example, MEX-3 is required for full translational repression of PAL-1, NOS-2, GLP-1, and ZIF-1 in anterior blastomeres [23, 55–57], and POS-1 and GLD-1 account for translational repression of key transcripts in posterior blastomeres [24, 55, 58].

### Maternal degraded transcripts are over-represented among asymmetric transcripts

Maternally loaded mRNA transcripts of the *C*. *elegans* early embryo have been previously categorized into classes according to the dynamics of their distribution. Whereas Class I mRNAs are maintained in the embryo over time, Class II mRNAs are progressively degraded in somatic blastomeres. Known Class II mRNAs are limited to a few examples such as *nos-1* and *nos-2*, which degrade in somatic cells beginning at the 4-cell stage but co-localize with the P granules in the P lineage [59]. Baugh et al. identified 1749 genes that produced “Maternal Degradation” [MD] transcripts by microarray time-course assays on whole embryos. MD transcripts are hypothesized to contain Class II mRNAs as well mRNAs that are degraded in other spatial patterns [60].

**Fig 6:**
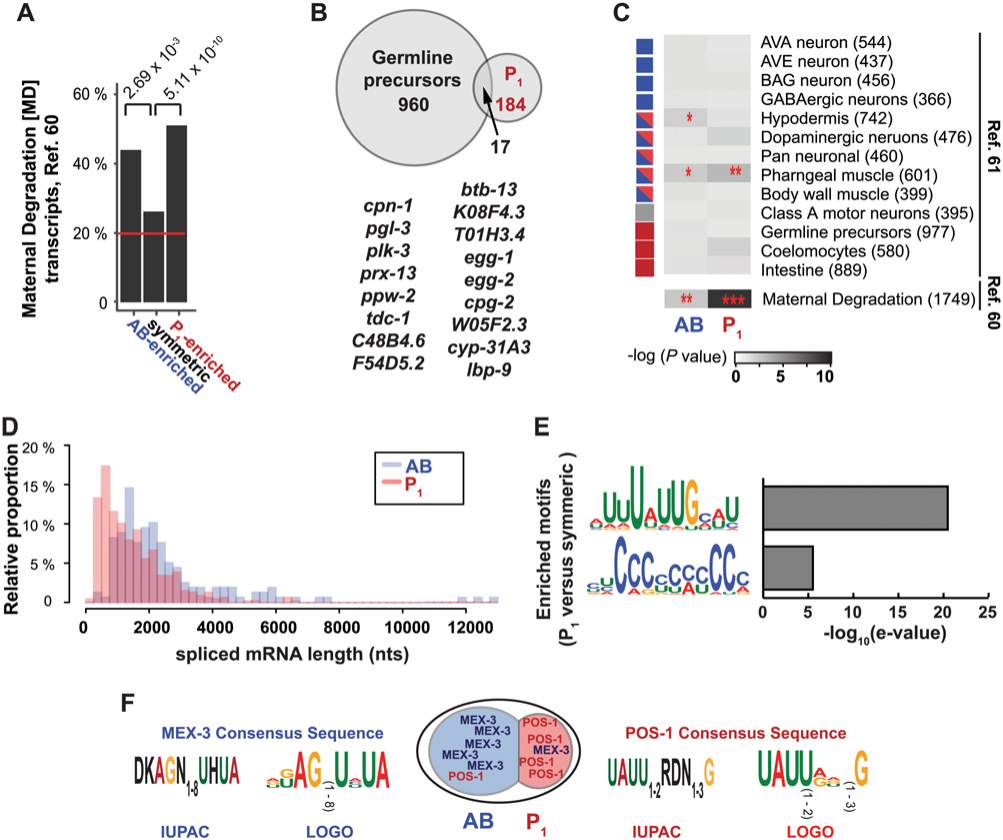
Characteristics of asymmetrically abundant genes. **(A)** The prevalence of Maternal Degradation [MD] transcripts (n = 1812) [60] among AB-enriched (n = 80), P_1_-enriched (n = 201), and symmetric transcripts (n = 2815). MD transcripts make up 19.8% of genes expressed at the 2-cell stage (horizontal red line). **(B)** Overlap between the P_1_-enriched gene set and transcripts enriched in Z_2_ and Z_3_ cells [61]. All individual genes that overlap are listed. **(C)** Association of cell-type specific transcripts [61] with the AB-enriched and P_1_-enriched gene sets. (*P < 5x10^-2^; **P < 5x10^-3^; ***P < 5x10^-9^, by Fisher’s exact test). Squares on the left indicate the lineage of origin for each tissue or cell type (blue = AB-lineage, blue and red = AB and P_1_ lineages, red = P_1_-lineage). For comparison, overlap between the MD transcripts (Fig 6A) is also plotted. **(D)** Lengths of spliced gene models for AB-enriched genes and P_1_-enriched genes plotted as a histogram. The *P* value for the likelihood that the two distributions were drawn from the same population was calculated using Wilcoxon rank-sum (Mann-Whitney *U*) test with continuity correction (*P* < 1.0x10^-16^ for all genes and *P* = 1.22x10^-15^ when the three outlying AB-enriched gene models were excluded). **(E)** Identification of *de novo* RNA sequence motifs specific to the 3′ UTRs of P_1_-enriched transcripts as compared to symmetric transcripts [62]. No significant motifs were identified between AB-enriched transcripts compared to symmetric transcripts. **(F)** POS-1 and MEX-3 proteins are known to be asymmetrically localized at the 2-cell stage of development. The consensus sequences required for POS-1 [63] and MEX-3 [64] recognition are shown as IUPAC codes and transformed into WEBLOGOs for ease of comparison with *de novo* logos (Fig 6E).

AB-enriched and P_1_-enriched transcripts were almost twice as likely to be categorized as MD maternal mRNAs when compared to symmetrically distributed transcripts (43.9% [26/66] of AB-enriched, 51.0% of P_1_ enriched [25/147], and 26.1% [737/2815] of symmetric transcripts were MD; Fig 6A–6C). This suggests that asymmetrically distributed transcripts were more likely to decrease in relative abundance as embryogenesis progressed compared to symmetrically distributed transcripts. This decline in abundance could be due to a uniform degradation of the transcript across the whole embryo or a relative reduction in the amount of transcript as it becomes restricted to a specific cell lineage. Indeed, by *in situ* hybridization (Fig 3H, S4 Fig) transcripts asymmetrically abundant in the P_1_ cell often exhibited P_2_-specific localization at the 4-cell stage.

Transcription is largely quiescent in the P_1_ cell and its descendants, so it is possible that maternal mRNAs are specifically sequestered in that lineage for later use in germ line development. The transcriptomes of the Z_2_/Z_3_ progenitor germ cells isolated from early embryos have been previously characterized by sorting them using *Ppie-1*::*GFP*::*PGL-1* fluorescent markers [61]. We did not find a statistically significant over-representation of P_1_-enriched transcripts among Z_2_/Z_3_-enriched transcripts (Fig 6B and 6C). This indicates that P_1_-enriched transcripts are not set aside for retention in the Z_2_/Z_3_ germ line precursors at a higher rate than those found in the AB-enriched or symmetric sets at the 2-cell stage. However, individual P_1_-enriched transcripts may be of particular interest in germ line precursor biology. Indeed, transcripts that are both enriched in P_1_ and in Z_2_/Z_3_ include *pgl-3*, which encodes one of the two key scaffolding proteins of the P granules [65] (Fig 6B), and *plk-3*, a component whose presence is correlated with P granule development [66].

To determine whether asymmetric transcripts were associated later in development with a specific cell or tissue type, we asked whether AB- or P_1_-enriched transcripts were over-represented in any specific embryonic cell type [61]. We found significant associations between AB transcripts and hypodermal transcripts, AB transcripts and pharyngeal transcripts, and between P_1_ transcripts and pharyngeal transcripts (Fig 6C, S6 Dataset). However, we saw no clear association between AB-enriched transcripts and cell types that were exclusive to the AB-lineage or between P_1_-enriched transcripts and cell types that were exclusive to the P_1_-lineage. This suggests that the primary purpose of asymmetric transcript abundance following the first embryonic division is *not* to retain transcripts for cells that arise later in each lineage.

Together these results suggest that mRNAs asymmetrically abundant at the 2-cell stage tend to decline rapidly in relative abundance as embryogenesis progresses, but that only a small fraction of the corresponding transcripts are tissue-specific or lineage-specific later in development. These findings are consistent with the observation that the transcriptome undergoes waves of change throughout development [60]. These waves are shaped through RNA degradation and zygotic transcription [67, 68].

### Distinct features and sequence content of asymmetric transcripts provide clues to potential mechanisms of asymmetric distribution

RNA-binding proteins recognize sequences and topologies in their RNA targets to affect localization, stabilization, polyadenylation, sequestration or degradation. We asked whether transcripts enriched in the AB or P_1_ cells shared RNA sequence features that could account for their patterning. Though we found no associations between 5′ splice leader usage, 5′ UTR length, 5′ UTR nucleotide composition, 3′ UTR length, or 3′ UTR sequence composition and cell-specific categories (S6A and S6B Fig), we found that short gene models (both spliced and unspliced) were associated with P_1_ enrichment, and longer gene models with AB-enrichment. The median AB-enriched transcript length was longer than the median length of symmetric transcripts, which in turn was longer than the median of P_1_-enriched transcripts (Fig 6D, S6C Fig). A large number of P_1_-enriched transcripts were very short (250–750 bp), a length class that was virtually non-existent in the AB set. Of the 73 genes whose models yielded spliced lengths of less than 750 bp and were P_1_-enriched, 13 were neuropeptide-like proteins (*nlp* and *flp*) and 14 were ribosomal protein components (*rpl* or *rps*).

We speculated that asymmetrically abundant transcripts might contain different RNA-binding protein associated sequence motifs or miRNA target sites. We searched *de novo* for RNA sequences that distinguished the AB-enriched or P_1_-enriched 3′ UTRs from those of the symmetric set. We identified two motifs over-represented in the 3′ UTRs of P_1_-enriched genes: UUUAUUGCAU and polyC (10-mer C) but none that were over-represented in the AB-enriched set of genes (Fig 6E) [62, 69, 70]. The UUUAUUGCAU motif is an Adenine and Uracil rich element (ARE) that contains features similar to known POS-1 and MEX-3 recognition sequences. The UAUU sequence is similar to the zinc-finger coordinated UAUU sequence found in the recognition sequence of the CCCH-type zinc finger protein POS-1 (UA(U_2-3_)RD(N_1-3_)G) and its human homolog, tristetraprolin (TPP-1, UAUUUAUU). The UUUAUUGCAU motif also resembles two stretches of UUUAUUGA within the *nos-2* 3′ UTR that are perfectly conserved in three *Caenorhabditis* species, are required for MEX-3 binding *in vitro*, and are required for preventing NOS-2 protein accumulation in anterior blastomeres [52, 56]. POS-1 and MEX-3 are two RNA binding proteins that are asymmetrically distributed in the 2-cell stage embryo and whose target sequence motifs have been described (Fig 6F). We noticed a striking similarity between the POS-1 target sequence and the P_1_-enriched sequence we identified (Fig 6E) suggesting that POS-1 or another CCCH-type zinc finger may function in the retention of P_1_-enriched transcripts.

## Discussion

### An expanded catalog of asymmetrically patterned mRNAs

We have expanded the knowledge of which mRNA molecules are asymmetrically distributed during the first cell division in *C*. *elegans*, a powerful model for asymmetric cell division and early development. Our approach led to the identification of *neg-1*. a gene that produces anterior mRNA and protein and is important for ventral enclosure and elongation, processes that involve hypodermal cells derived from the AB-lineage.

### *neg-1* is an anterior-enriched transcript that is important for anterior morphogenesis

We identified a transcript, *neg-1*, that is more abundant in the anterior cell following the first cell division of the *C*. *elegans* zygote. The protein product of this transcript is also preferentially localized to anterior nuclei, starting in the following cell division. Disruption of *neg-1* by RNAi led to defective anterior morphogenesis due to failures in hypodermal cell ventral enclosure and elongation. The hypodermal leader cells (ABpraappap, ABpraapppa, ABplaappap, and ABplaapppa), the ventral cells posterior to the leader cells (ABpraapppp, ABplaapppp, and the P cells), and the seam cells (H0, H1, V1–V6, and T) are all derived from the AB lineage and are all actively involved in hypodermal cell enclosure along the ventral midline. The C founder cell yields other hypodermal cells but these are posterior and dorsally located and do not play as large a role in ventral enclosure [8, 71]. Thus, the data presented here show that the asymmetrically abundant *neg-1* mRNA is associated with subsequent asymmetric protein abundance, and that loss of *neg-1* function has consequences on anterior morphogenesis. It remains an open question as to how a protein whose anterior patterning is most striking at the 4-cell stage yields an RNAi phenotype 300 minutes later. NEG-1 may alter or regulate gene expression in the AB lineage, which would be consistent with its positive charge and concentration in the nucleus.

### Asymmetric transcripts are associated with biological processes that differentiate AB and P_1_ lineages

The functional characterization of *neg-1* in this manuscript indicates that cell-specific RNA profiling may be a fruitful way to identify transcripts and proteins that contribute to lineage-specific processes. We identified *neg-1* based on its preferential abundance in the AB cell. With this in mind, other transcripts identified in our study warrant special attention with respect to their potential for lineage-specific functions. For example, *cdc-25*.*3* was AB-enriched. Its homolog *cdc-25*.*1* regulates relative cell-cycle rate in other lineages [9]. *cdc-25*.*1* and *cdc-25*.*3* may work together to regulate lineage-dependent cell-cycle rates. Many of the AB-enriched transcripts, such as *erm-1* and *hmp-2*, are associated with epithelial adherens junctions, possibly foreshadowing the important role of hypodermal cell function among the progenitors of the AB lineage. Cytoskeletal regulatory genes enriched in the AB lineage such as *cyk-1* and *dnc-1* may indicate the requirement for greater cytoskeletal control in the AB lineage.

RNAs enriched in the P_1_ cell also function in processes known to be important to the P lineage. One such transcript encodes PGL-3, the protein that comprises the scaffold for P granules. Further, several RNAs encoding ribosomal protein genes and RBPs important for translation were over-represented in the P_1_ cell. This is consistent with the differential translation of specific mRNAs (*glp-1*, *pal-1*, and *nos-*2) in the anterior and posterior blastomeres that generates proteomic diversity prior to the onset of zygotic transcription [52, 57, 72]. Because key RBPs that regulate those mRNAs also bind other targets, cohorts of mRNAs may be coordinately regulated [73, 74]. The process of translation in general may be distinct between the P lineage and somatic lineages because P granules and germ granules contain mRNAs and proteins, such as IFE-1, that regulate general aspects of translational biology [75]. Future studies may address how P_1_-enriched transcripts associated with translation impact protein production in that lineage.

### Asymmetric distribution of RNA molecules as a mechanism for cell fate determination

The search for asymmetrically partitioned cell-fate determinants has traditionally concentrated on differential gene expression patterns, which can arise through both transcriptional differences and differential post-transcriptional regulation of mRNAs. For example, *ASH1* mRNA localizes to *Saccharomyces cerevisiae* bud cells and marks bud-versus-mother cell identity [76, 77], and the mRNA transcripts of *gurken*, *bicoid*, *oskar*, and *nanos* distribute asymmetrically in the *Drosophila melanogaster* embryo and are required for embryonic patterning (recently reviewed in [78] and [79]). The concentration of a particular mRNA transcript in a specific lineage can also be important for lineage-related functions even if it is not acting to determine the identity of that lineage directly.

Our study identified an asymmetrically distributed mRNA transcript, *neg-1*, which was important for morphogenesis. However, we have yet to establish whether *neg-1* is a cell fate determinant or whether AB-enrichment of *neg-1* mRNA is required for its function for embryogenesis. The AB-enrichment of *neg-1* mRNA preceded an anterior enrichment of NEG-1 protein by one cell division, but it remains possible that the asymmetric RNA localization is not required for asymmetric NEG-1 protein localization. For example, translational control could be the primary mechanism shaping the proteomes of daughter cells. Translation of specific transcripts is regulated during oogenesis, early embryonic development, and in the germ cell lineage. It could be that the asymmetric mRNAs we observe arise as a downstream effect of translational control. Even if so, cataloging asymmetrically abundant mRNAs is likely to be an efficient way to identify genes that are spatially regulated and important for development.

### How do mRNAs become asymmetrically abundant?

The initial symmetry of the round oocyte is broken by the ellipsoid shape of the fertilized egg and by the entry point of the sperm centrosome [7, 80, 81]. The PAR proteins transduce those polarity signals to create a polarized actinomyosin network, directional cytoplasmic flow, and a skewed arrangement of the mitotic spindle [82–86]. As a result, the division plane of first mitosis in the *C*. *elegans* zygote is situated closer to the posterior. This process involves progressive stages of polarity initiation, maintenance, and amplification. How is mRNA location and abundance regulated during this process? While a variety of mechanisms, including differential diffusion, stability, degradation, localization, sequestration, or polyadenylation may be regulating the RNA abundance patterns we identified in our study, our data provided two clues regarding where to look next.

First, we observed that AB-enriched transcripts were significantly longer than symmetric transcripts, and that these were, in turn, longer than P_1_-enriched transcripts. It is possible the shorter length of the P_1_-transcripts decreases the likelihood that they will contain miRNA binding sites or RNA-Binding Protein (RBP) target sequences, allowing for preservation of the shorter mRNAs in the P_1_ cell. Alternatively, the shorter 3′ UTRs may lack motifs that direct or stabilize transcripts in the AB cell. Finally, it is possible that bulk cytoplasmic flow within the zygote passively accumulates smaller transcripts in the posterior end of the cell, whereas longer transcripts are retained in the anterior side. Given that the actinomyosin network is known to adopt asymmetric qualities after fertilization [84] and given that poroelastic diffusion properties of the cytoplasm are highly dependent on actiomyosin [87], passive diffusion may contribute to differential distribution of molecules in the early embryo.

Second, we identified an AU-riche element (UUUAUUGCAU) that was over-represented among the P_1_-enriched transcripts. This motif bears striking resemblance to the recognition motif of POS-1 (UA(U_2-3_)RD(N_1-3_)G) but also bears similarity to the direct repeats (called DR1 and DR2) recognized by MEX-3 within the *nos-2* 3′ UTR (UUUAUUGA) [63, 74]. In general, AU-rich elements are associated with mRNA stability and decay via RBPs. Our identified sequence motif could be a MEX-3 recognition motif, a degenerate POS-1 motif, the binding motif for another RBP such as MEX5/6, or a motif that is bound combinatorially by multiple RBPs. *nos-2* is translationally repressed in anterior blastomeres in a MEX-3-dependent manner [56], and *nos-2* mRNA is degraded in anterior blastomeres in a MEX-5/MEX-6-dependent manner [52]. It is possible that multiple P_1_-enriched transcripts and their protein products are similarly restricted from anterior blastomeres. Alternatively, POS-1 or some other RBP may recognize this degenerate motif to sequester transcripts in the P_1_ cell.

In addition to these two observations derived from the bulk behavior of asymmetric transcripts in our RNA-seq approach, our smFISH observations also provide some guidance regarding mechanism. The transcripts *chs-1* and *bpl-1* were uniformly distributed in 1-cell embryos just before division and apparently became asymmetrically abundant after division occurred. In addition, the asymmetry of the *pgl-3* transcript seemed more dramatic in late 2-cell stage embryos compared to early 2-cell embryos. This suggests that mechanisms responsible for asymmetric abundance of some mRNA transcripts are not wholly dependent on differential trafficking prior to cell division. Differential transcript stabilization or degradation may be responsible for generating cell-specific mRNA patterns throughout the cell cycle. We identified a PolyC sequence over-represented in the 3′ UTRs of P_1_-enriched transcripts. Recently, PolyC motifs were shown to be required for a wave of post-fertilization RNA degradation during the oocyte-to-embryo transition [88]. It is possible that the P_1_-lineage may be more resistant to this degradation than the AB-lineage.

The smFISH experiments also revealed that *chs-1* and *bpl-1* transcripts were present in larger granular particles. Despite the superficial resemblance to P granules, we were unable to determine whether these structures were P granules. Unlike P granules, the *chs-1* and *bpl-1* transcript granules did not appear to coalesce or posteriorly localize during prophase of the 1-cell stage embryo. It is possible that granular association promotes P_1_-enrichment of transcripts through sequestration or stabilization.

Asymmetric cell divisions are commonplace in development and influence fate decisions in systems ranging from plant stomata to cancer stem cells. The *C*. *elegans* early embryo is unique in the reproducibility of division timings and planes, the highly deterministic nature of the resulting lineages, and the lack of zygotic transcription in the earliest divisions. This study determined the mRNA abundance in each of the two cells created after the first zygotic cell division. This led to the identification of a new gene important for lineage-specific functions and clues regarding the post-transcriptional mechanisms that control cellular mRNA abundance in early embryogenesis.

## Materials and Methods

### Worm strains and isolations

*N2* (wild type) worm strains were obtained from the *Caenorhabditis* Genome Center and maintained at 20°C unless otherwise indicated using standard protocols [89]. *dlg-1*::*GFP (FT64)* was obtained from the CGC.

### Blastomere dissection

Blastomere isolations were performed by embryo isolation, eggshell degradation and micromanipulated separation as previously described [90]. We used chitinase from *Streptomyces griseus* (sigma, C6137) at 10 U/ml due to the recent unavailability of *Serratia marcascens* chitinase. Isolated AB and P_1_ cells were dropped directly into Trizol solution and stored at -80°C if not immediately processed.

### RNA isolation

Blastomere preparations were thawed and re-frozen up to three cycles to lyse them. RNA extraction was performed using a Trizol/chloroform extraction followed by RNeasy Micro (Qiagen) preparation using On Column DNaseI Digestion (Qiagen).

### mRNA amplification and sequencing

RNA amplification was performed by *in vitro* transcription of an oligo dT-primer fused T7 promoter producing up to a 10,000-fold increase in mRNA using one round of a TargetAmp aRNA Amplification Kit (Epicentre). The resulting *in vitro* amplified RNA was used for a standard RNA-seq protocol (Illumina mRNA Sequencing Sample Preparation Guide, September 2009). Magna beads were used for purification steps and for the final purification. Gel extraction was not necessary due to the size of *in vitro* amplified RNA fragments. Samples were sequenced on either GA-2 or Hi-Seq Illumina sequencers with either 36 or 50 rounds of sequencing. Data from this study are available on NCBI GEO (GSE59943)

Reviewer access: http://www.ncbi.nlm.nih.gov/geo/query/acc.cgi?acc=GSE59943

### Comparison between mRNA amplification and sequencing to traditional mRNA-seq

We sequenced 5 ug of unamplified RNA by the standard Illumina RNA-seq methodology. The same RNA sample was diluted down to 500 pg and sequenced using RNAamplification and sequencing.

### RNA-seq analysis

Sequencing reads were filtered for primer and adapter sequences [91] and a minimal quality score of 20 using Tagdust (Version 1.12) [91], aligned to the *C*. *elegans* ce10 genome using Tophat (1.4.0) [30]. The resulting alignment files were quantified using HT-Seq (0.5.3) [33] and the RefSeq gene annotation for WS220 downloaded March 23, 2012 and reads were assigned per gene model. The significance of differentially represented transcripts were quantified by DESeq (1.2.1) [32] in R 2.12 [92] using size estimation normalization.

### Differential expression analysis

Reads were called as present if the number of mapped reads met a minimum 50 reads in the mean of all samples and at least 1 read in the mean of either AB or P_1_ samples. We identified 7945 present genes of 20,240 of the predicted gene models. Present genes were called as giving rise to significantly asymmetric transcripts if they met a *P* value < 0.1 after FDR adjustment using the Benjamani-Hochberg method within the DEseq package (1.2.1) [32] in R 2.12 [92] (All other R analyses were performed in 3.0). When necessary, genes were ranked by negative log *P* value.

### Comparison to publicly available in situ hybridization images

The 80 AB-enriched transcripts and 201 P_1_-enriched transcripts identified by RNA-seq were surveyed for entries in a publicly available database of RNA *in situ* hybridization images [34]. A subset of 80 (of 7664) symmetric transcripts were selected as a comparison. These 80 were the middle-most symmetric transcripts as ranked by log_2_ P_1_/AB fold change. Many transcripts were absent from the online database, showed no staining, or were uninterpretable for other reasons. Entries that had successful staining were scored in a blind survey we constructed. Multiple reviewers categorized sets of images from each entry as either “AB”, “Marginal AB”, “Symmetric”, “Marginal P_1_-biased”, P_1_”, “No expression”, or “Ambiguous”. The rubric for these categories is included as S1 Text. The full dataset including each reviewer’s response, the combined overall score, and links to publicly available images [34, 93] is included as S3 Dataset.

### qRT-PCR

Pools of five P0, AB, and P_1_ blastomeres were isolated using dissection methods above and immediately processed using the CellsDirect cDNA kit (Invitrogen). The abundance of mRNA transcripts was quantified using the SYBR Maxima qPCR system (Thermo Scientific) on an ABI 7900HT Fast Real Time PCR System using a dilution series to calibrate relative abundance of each transcript. Only transcripts with an abundance over 15,000 mean reads passed the requirement of generating a linear standard dilution curve over a 1–1/1000 fold dilution series. Primers were designed to lie within the 3′ most exon and within 200 bp of the terminal codon. Each gene reported represents at least 2 independent biological samples, each of which was measured in 3 technical replicates; means of sample means and standard error of the mean were calculated.

### smFISH

Stellaris smFISH fluorescent probe sets (Biosearch) were generated to hybridize to *mex-3 neg-1*, *tes-1*, *chs-1*, *pgl-3*, *bpl-1*, *B0495*.*7*, *set-3*, and *gpd-2* (S2 Text). N2 worms were grown at 20°C to gravidity on NGM plates, bleached for embryos, resuspended in -20°C methanol, freeze cracked in liquid nitrogen, and fixed at -20°C for 2–48 hours. A protocol that combined Shaffer et al. [94] and Ji et al. [95] was performed. Embryos were equilibrated in WB1 (100 mg/ml dextran sulfate, 10% formamide, 2 x SSC), hybridized in hybridization buffer (100 mg/ml dextran sulfate, 1 mg/ml *E*. *Coli* tRNA, 2 mM vanadyl ribonucleoside complex, 0.2 mg/ml BSA) containing 50 pmoles of each primer set. Hybridization at 30°C overnight was followed by two WB1 washes, DAPI staining, and three 2 x SSC washes. Embryos were mounted as described in Ji et al. [95] using SlowFade (Life Technologies) to prevent photobleaching.

All smFISH images were acquired using a Cool Snap HQ2 camera on a DeltaVision-modified inverted microscope (IX71; Olympus), with a UPlanSApo 100 x (1.40 NA) objective and SoftWorx software (Applied Precision) using fixed exposure and acquisition conditions (0.3 μm z-stacks, up to three wavelengths). Images were deconvoluted. Quantification was performed using specialized scripts in ImageJ and R to identify thresholded particles, their pixel intensities, and their cell boundaries. The number of discrete particles was quantified and normalized to cell area (proportional to volume because cells are flattened). The total internal intensity of each particle was also measured, and the background-subtracted fluorescence was normalized to cell area. For statistical tests, two probes were multiplexed within the same embryo and Wilcoxon signed-rank tests were performed on the paired ratios.

### RNAi feeding and embryonic lethality phenotyping

dsRNA feeding was carried out as previously described [96] by moving worms to RNAi feeding plates at L4 stages of development. 24 hours and 48 hours after feeding, worms were assessed for their ability to lay viable embryos by capturing all eggs laid within the next 24-hours and scoring for hatching and viability 48–72 hours later. For each strain queried, we performed 2–3 replicates in which 4–6 mothers were assayed at both the 24- and 48-hour time points.

### RNAi injection and 3D microscopy

dsRNA was produced using the T7 RiboMAX Expression System (Promega, P1700). RNA was injected into L4 stage worms as previously described [96, 97]. Images were acquired 24 hours post-injection for injected, uninjected, and mock-injected worms. Experiments were carried out at 20°C unless otherwise noted. RNAi knockdown and control worms were imaged on the same slide. Time-lapse images collected in Fig 5C, were acquired using Nomarski DIC optics on a Nikon Eclipse E800 using a Nikon 1.40 NA 60x objective as previously described in Dickinson et al. [98]. Images were collected every 2 minutes over 9 hours with z-stacks at 1 μm intervals for a total depth of 30 μm. For Fig 5D, images were captured on a spinning disc CSU-XI Yokogawa confocal system mounted on an inverted microscope (Eclipse Ti, Nikon), 16-bit cooled CCD camera (Image E, Hamamatsu), 50 mW air-cooled Arbon laser (Laser Physics), Nikon 1.4 NA 60x objective, and Metamorph Software. Images were captured every 15 min over 9 hours with z-stacks at 3 μm intervals for a total depth of 22 μm. Embryos were illuminated at 488 nm at 25% power, 500 ms, 1x1 bin for GFP settings and 100 ms, 1 x 1 bin for DIC settings.

### *mNEONGREEN*::*NEG-1*::*NEG-1-3′UTR* transgenic worms

We fused the *mex-5* promoter driving *mneongreen* (Allele Biotechnology) in frame, to the *neg-1* gene beginning at its start site and containing its full 3’ UTR plus 100 base pairs downstream. This was cloned into a hygromyocin selectable MOSSCI plasmid using a pCFJ150 backbone [99] constructed using Gibson Assembly (New England BioLabs). The resulting *mex-5promoter*::*neongreen*::*neg-1*::*neg-1-3’UTR* plasmid was transformed into *N2* worms, selected with hygromycin, and counter selected for single copy insertion using the *peel-1* system. NEONGREEN::NEG-1 fluorescence was imaged on an inverted microscope (IX71; Olympus), with an 100 x objective, and using 0.3 μm sections. Max intensity projections are shown.

### Gene ontology analysis

The list of present genes were separated by fold change and ranked by FDR adjusted *P* value as calculated in DESeq to produce a ranked list of AB-enriched genes and P_1_-enriched genes. Ranked lists were processed as input by the GOrilla algorithm and a false discovery rate-adjusted *P* value of 10^-5^ was set as a minimum cutoff [50]. GO terms and scores were summarized in REViGO to merge excessively overlapping terms [51]. Categories were filtered for those with frequencies of less that 5% of the genome and the REViGO adjusted log_10_ *P* values greater than 7 were plotted.

### Comparisons with publically available transcriptome datasets

The intersection between Maternal Degraded (MD) mRNAs [60] and AB-enriched, P_1_-enriched, and symmetric genes was calculated. Symmetric genes were defined as the 50% of present genes with the largest *P* values, as calculated in DEseq. The association between maternal mRNA category and differential abundance category was calculated using Fisher’s exact test. The intersection between tissue-specific transcriptomes [61] and AB-enriched, P_1_-enriched, and symmetric sets were also calculated. The association between representation and category was calculated using Fisher’s exact test between symmetric and AB-enriched or symmetric and P_1_-enriched categories.

### RNA feature characterization and analysis

Splice leader (SL) sequences, 5′ UTR sequences, spliced gene models, unspliced gene models, and 3′ UTR sequences were obtained from Mangone et al. [100] and Wormbase (November 27, 2013). To avoid redundancy in pools of sequences, only the longest 3′ UTR from the union set of multiple 3′ UTR models identified in both Mangone et. al. and Wormbase annotations were used. Association between cell-type category (AB-enriched, P_1_-enriched, symmetric) and gene feature were calculated using Fisher’s exact test for discrete data and Wilcoxon rank-sum (Mann-Whitney *U*) test for continuous data.

### *De novo* RNA motif searching

*De novo* motif searching was carried out using MEME (4.9.0). We sought discriminating motifs 5–12 nucleotides in length that distinguished 3′ UTR sequences in the AB-enriched or the P_1_-enriched set from the symmetric set focusing on the sense strand. 3′ UTR gene models were identified as the overlap between Mangone et al. [100] and Wormbase (November 27, 2013). The search model [–mod zoops] was used.

### Accession numbers

High throughput sequencing data was submitted to NCBI Geo as **GSE59943** (http://www.ncbi.nlm.nih.gov/geo/query/acc.cgi?acc=GSE59943). Raw sequences are available as **SRP/SRP045/SRP045110**. **GSE59943_ABP1_Nishimura_raw_matrix.txt.gz** reports the number of raw read counts for each gene model, tabulated for 20,240 genes. **GSE59943_ABP1_Nishimura_normalized_matrix.txt.gz** reports the number of size-normalized read counts for each gene model, tabulated for 20,240 genes. **GSE59943_AB_average.wig.gz** is a wig file that averages three scaled AB samples. This file is suitable for upload to UCSC genome browser. **GSE59943_P1_average.wig.gz** is a wig file that averages three scaled P_1_ samples. This file is suitable for upload to UCSC genome browser.

## Supporting Information

S1 Fig Validation of the RNA amplification and sequencing protocol. **(A)** To compare the RNA amplification and sequencing (RNA-amp-seq) approach for low-input samples against standard RNA-seq performed on high-input samples, we compared the two techniques on the same total RNA samples. A representative correlation plot between RNA-seq and RNA-amp-seq for one of two performed replicates is shown. **(B)** Two samples of embryo preparations collected at two time points, were sequenced using both RNA-seq and RNA-amp-seq. The genes over-represented in mid-stage embryos as compared to early embryos as assessed by RNA-seq are colored in red in both plots. The plot on the right was generated using measurements from the RNA-amp-seq method, illustrating the same genes shown to be enriched by RNA-seq also show an enrichment with the amplification method (red). The transcripts that were not identified as over-represented by the RNA-amp-seq technique (false negatives) are also colored (green). **(C)** The same as for S1B Fig but showing transcripts with higher abundance in early embryos as compared to mid-stage embryos with true positives (red) and false negatives (green) noted.
(EPS)

S2 Fig Pooling blastomeres yields RNA-seq datasets with lower variance relative to single-cell methods [16]. **(A)** To measure variance, coefficients of variance were calculated on the basis of normalized read counts obtained in our study and on the basis of absolute mRNA molecules per cell as measured in Hashimshony et al. The variances of each gene as produced in each study are plotted against one another. The x = y identity (red solid line) and two-fold change intevals (red dotted lines) are depicted. **(B)** Using normalized read count measurements obtained in this study, we plotted AB over P_1_ ratios against mean intensity for each transcript (same plot as Fig 1C). The asymmetric transcripts identified by Hashimshony et al. are highlighted in blue (AB-enriched) and red (P_1_-enriched) to illustrate their behaviors in our study (transcripts identified in our study are shown in Fig 1c). **(C)** The overlap between the genes identified by Hashimshony et al. and our study.
(EPS)

S3 Fig *in situ* hybridization images of AB-enriched, P_1_-enriched and symmetric transcripts. AB-enriched transcripts that yielded in situ patterns with scores of 2 or greater in a blind survey (plotted in Fig 2B) are shown. P_1_-enriched transcripts that yielded patterns that scored -2 or less are shown. A subset of symmetric transcripts are also shown. The anterior AB cell is always oriented to the left of the posterior P_1_ cell. Red arrowheads indicate the cell with higher observed signal. All images in B, C, and D were taken from the Nematode Expression Data Base (http://nematode.lab.nig.ac.jp/db2/index.php).
(EPS)

S4 Fig smFISH quantification of AB and P_1_ transcripts. *chs-1* and *bpl-1* but also *pgl-3* showed some signs of particle association into larger granules that could complicate quantification by simple particle count. As a complementary approach, we measured and quantified the cell volume normalized fluorescence intensity (summed) within particles and illustrate their quantities here (**B)** in comparison with the particle density measurements **(A)** that are also depicted in Fig 3.
(EPS)

S5 Fig *pgl-3* associates with posterior cells through early embryonic stages. *pgl-3* hybridization signals by smFISH microscopy are shown from 1-cell to the roughly 22-cell stage of development.
(TIF)

S6 Fig Sequence features associated with asymmetrically abundant transcripts. **(A)** We searched for gene features that distinguished the AB-enriched genes from the symmetric set of genes and the P_1_-enriched genes from the symmetric set of genes. Sequence, length, and characteristics of 5′ UTRs (Wormbase), 3′ UTR annotations (Wormbase, Mangone et al. [100]). splice leader usage (Mangone et al.), and spliced and unspliced gene model lengths were compared among the three gene sets. No statistically significant associations were found except gene model length (both spliced and unspliced). **(B)** Nucleotide frequencies in the entire transcript (spliced model) are shown. **(C)** The relative proportion of spliced mRNA gene model lengths (that ranged from 0–8000 nts) are shown for P_1_-enriched genes and symmetric genes. AB-enriched genes and symmetric genes are plotted in the right panel. *P* values for the likelihood that the two distributions were drawn from the same population were calculated using Wilcoxon rank-sum (Mann Whitney *U*) test with continuity correction (*P* = 1.364x10^-13^ for AB versus symmetric and *P* = 9.827x10^-9^ for P_1_ versus symmetric).
(EPS)

S1 Table AB-enriched transcripts and P_1_-enriched transcripts. A very simple list of the AB-enriched and P_1_-enriched transcripts as identified in this study.
(DOCX)

S1 Dataset AB and P_1_ transcriptome dataset. An excel file with the AB and P_1_ values and scores for all 20,240 genes. This file contains several worksheets. (1) Annotation data. This worksheet lists each column heading and its meaning. It also contains R-code that was used to filter and rank the lists. (2) Full Dataset. This worksheet lists raw count reads per gene for each sample, normalized count reads per gene for each sample, DESeq output analysis, and annotations of each gene. (2) Present subset. This is the same information from the Full Dataset but includes only genes that are ‘present’. (3) AB subset. This is the same information from the Full Dataset but includes only genes that were identified as AB-enriched. (4) P_1_ subset. This is the same information from the Full Dataset but includes only genes that were identified as P_1_-enriched. (5) Symmetric subset. This is the same information from the Full Dataset but includes only genes that were identified as symmetric. Data from these spreadsheets was used to generate Fig 1C, the RNA-seq fold-change in Fig 2E, and S2B Fig.
(XLSX)

S2 Dataset Public *in situ* hybridization data. An excel file containing three spreadsheets documenting the transcripts we queried in the Kohara database, and their patterns of *in situ* hybridization. Data from this file was used to generate Fig 2A and 2B.
(XLSX)

S3 Dataset qRT-PCR documentation. An excel file containing measurements for the qRT-PCR experiments. Data from this file was used to generate Fig 2C.
(XLSX)

S4 Dataset Embryonic lethality RNAi. An excel file containing percentage embryonic lethality rates identified for a subset of AB-enriched and P_1_-enriched genes. Data from this file was used to generate Fig 4A.
(XLSX)

S5 Dataset Gene ontology. An excel file containing GO ontology ID’s over-represented in the AB-enriched and P_1_-enriched list. It contains GO terms, REViGO output, and the genes that contribute to each category. Data from this file was used to generate Fig 5.
(XLSX)

S6 Dataset Comparison to published gene sets. An excel file containing a list of all genes in the 7945 present genes. This file tabulates those genes that occurred in the list of Maternal Degraded [MD] mRNAs [60] and those that occurred in tissue-type enriched datasets [61]. Data from this file was used to generate Fig 6A–6C.
(XLSX)

S7 Dataset Coefficient of Variance. An excel file that contains the size-normalized counts merged from this paper and from Hashimshony et al. Coefficient of Variance calculations and a list of the overlapping genes are included. Data from this file were used to generate S2A Fig and S2C Fig
(XLSX)

S1 Text Rubric for scoring Public *in situ* hybridization data. A pdf document listing the rubric used to instruct reviewers how to score the transcripts were queried in the Kohara database and their patterns of in situ hybridization at the 2-cell stage of development.
(PDF)

S2 Text smFISH probe sets. A text file containing the smFISH probes used in this study. Probes were designed by Stellaris. Probes documented in this file were used to generate hybridization images shown in Fig 3, Fig 5, S4 Fig, and S5 Fig.
(TXT)

S3 Text Quantitative microscopy dataset. A text file containing the number of particles and internal fluorescence counts for each embryo imaged by the smFISH probes listed in S2 Text. Analyzed data from this raw set is illustrated in Fig 3 and S4 Fig.
(TXT)

S4 Text PSPM of Motif 1. A text file containing the Position Specific Probability Matrix (PSPM) identified by MEME. This motif was over-represented in the 3′ UTR sequences of P_1_-enriched transcripts relative to the set of 3′ UTR sequences of symmetric transcripts.
(TXT)

S5 Text PSPM of Motif 2. A text file containing the PSPM identified by MEME. This motif was over-represented in the 3′ UTR sequences of P_1_-enriched transcripts relative to the set of 3′ UTR sequences of symmetric transcripts.
(TXT)

S6 Text RNA dilution experiments. A text file containing raw and normalized reads for a study in which samples of total RNA were profiled with both the low input RNA-amp-seq protocol and the standard RNA-seq protocol. Data from this file were used to generate S1A–S1C Fig.
(TXT)
