# Supplementary figures and images for: Asymmetric Transcript Discovery by RNA-seq in C. elegans Blastomeres Identifies neg-1, a Gene Important for Anterior Morphogenesis
Source: PLoS Genet. 2015 Apr 13;11(4):e1005117. doi: 10.1371/journal.pgen.1005117 (PMC4395330; doi:10.1371/journal.pgen.1005117)

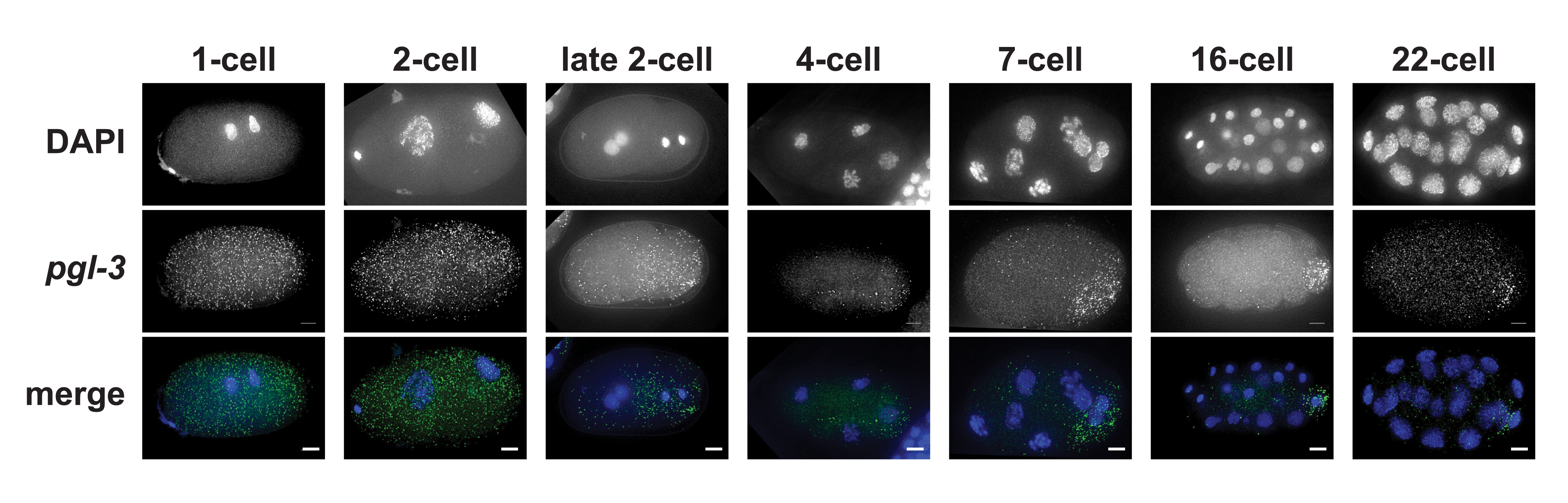

Supplement: S5 Fig — pgl-3 hybridization signals by smFISH microscopy are shown from 1-cell to the roughly 22-cell stage of development. (TIF) [file pgen.1005117.s005.tif]
