# Supplementary material for: Asymmetric Transcript Discovery by RNA-seq in C. elegans Blastomeres Identifies neg-1, a Gene Important for Anterior Morphogenesis
Source: PLoS Genet. 2015 Apr 13;11(4):e1005117. doi: 10.1371/journal.pgen.1005117 (PMC4395330; doi:10.1371/journal.pgen.1005117)
Supplement: S1 Table — A very simple list of the AB-enriched and P1-enriched transcripts as identified in this study. (DOCX) [file pgen.1005117.s007.docx]

| **Rank** | **Cell** | **WBID** | **Public name** | **Sequence**  **name** | **Refseq name** |
| --- | --- | --- | --- | --- | --- |
| 1 | AB | WBGene00001333 | erm-1 | C01G8.5 | erm-1 |
| 2 | AB | WBGene00013481 | Y69H2.3 | Y69H2.3 | Y69H2.3 |
| 3 | AB | WBGene00016824 | C50E3.13 | C50E3.13 | C50E3.13 |
| 4 | AB | WBGene00017985 | F32D1.6 | F32D1.6 | F32D1.6 |
| 5 | AB | WBGene00021009 | afd-1 | W03F11.6 | afd-1 |
| 6 | AB | WBGene00009606 | F40G12.11 | F40G12.11 | F40G12.11 |
| 7 | AB | WBGene00002276 | lem-3 | F42H11.2 | lem-3 |
| 8 | AB | WBGene00016823 | C50E3.12 | C50E3.12 | C50E3.12 |
| 9 | AB | WBGene00000388 | cdc-25.3 | ZK637.11 | cdc-25.3 |
| 10 | AB | WBGene00020948 | W02F12.3 | W02F12.3 | W02F12.3 |
| 11 | AB | WBGene00009921 | F52B5.2 | F52B5.2 | F52B5.2 |
| 12 | AB | WBGene00001898 | his-24 | M163.3 | his-24 |
| 13 | AB | WBGene00008080 | C44B9.3 | C44B9.3 | C44B9.3 |
| 14 | AB | WBGene00016955 | C55C3.5 | C55C3.5 | C55C3.5 |
| 15 | AB | WBGene00008823 | F14H3.4 | F14H3.4 | F14H3.4 |
| 16 | AB | WBGene00011153 | R09A8.2 | R09A8.2 | R09A8.2 |
| 17 | AB | WBGene00013981 | ZK507.6 | ZK507.6 | ZK507.6 |
| 18 | AB | WBGene00020375 | T09B4.1 | T09B4.1 | T09B4.1 |
| 19 | AB | WBGene00017755 | F23F12.9 | F23F12.9 | F23F12.9 |
| 20 | AB | WBGene00009793 | pkn-1 | F46F6.2 | F46F6.2 |
| 21 | AB | WBGene00008775 | F13H10.4 | F13H10.4 | F13H10.4 |
| 22 | AB | WBGene00008464 | E02H4.6 | E02H4.6 | E02H4.6 |
| 23 | AB | WBGene00000090 | age-1 | B0334.8 | age-1 |
| 24 | AB | WBGene00018009 | F33D11.12 | F33D11.12 | F33D11.12 |
| 25 | AB | WBGene00008789 | F14D7.2 | F14D7.2 | F14D7.2 |
| 26 | AB | WBGene00000146 | ape-1 | F46F3.4 | ape-1 |
| 27 | AB | WBGene00019792 | M116.5 | M116.5 | M116.5 |
| 28 | AB | WBGene00008825 | F14H3.6 | F14H3.6 | F14H3.6 |
| 29 | AB | WBGene00015924 | C17H11.4 | C17H11.4 | C17H11.4 |
| 30 | AB | WBGene00016812 | C50D2.9 | C50D2.9 | C50D2.9 |
| 31 | AB | WBGene00020269 | T05H4.6a | T05H4.6 | T05H4.6 |
| 32 | AB | WBGene00011883 | T21B10.1 | T21B10.1 | T21B10.1 |
| 33 | AB | WBGene00017263 | F08F3.6 | F08F3.6 | F08F3.6 |
| 34 | AB | WBGene00021467 | Y39G10AR.9 | Y39G10AR.9 | Y39G10AR.9 |
| 35 | AB | WBGene00008219 | C50B6.3 | C50B6.3 | C50B6.3 |
| 36 | AB | WBGene00000872 | cyk-1 | F11H8.4 | cyk-1 |
| 37 | AB | WBGene00012385 | Y5F2A.4 | Y5F2A.4 | Y5F2A.4 |
| 38 | AB | WBGene00008535 | F02H6.2 | F02H6.2 | F02H6.2 |
| 39 | AB | WBGene00001949 | hlh-2 | M05B5.5 | hlh-2 |
| 40 | AB | WBGene00008876 | F16A11.1 | F16A11.1 | F16A11.1 |
| 41 | AB | WBGene00010502 | K02C4.3 | K02C4.3 | K02C4.3 |
| 42 | AB | WBGene00003229 | mex-3 | F53G12.5 | mex-3 |
| 43 | AB | WBGene00022534 | syn-16 | ZC155.7 | syn-16 |
| 44 | AB | WBGene00009065 | F22G12.5 | F22G12.5 | F22G12.5 |
| 45 | AB | WBGene00020168 | T02G5.12 | T02G5.12 | T02G5.12 |
| 46 | AB | WBGene00021468 | epg-2 | Y39G10AR.10 | Y39G10AR.10 |
| 47 | AB | WBGene00011432 | sdz-30 | T04D3.2 | sdz-30 |
| 48 | AB | WBGene00017037 | D1069.3 | D1069.3 | D1069.3 |
| 49 | AB | WBGene00010284 | aman-2 | F58H1.1 | aman-2 |
| 50 | AB | WBGene00000161 | apa-2 | T20B5.1 | apa-2 |
| 51 | AB | WBGene00021636 | pcaf-1 | Y47G6A.6 | pcaf-1 |
| 52 | AB | WBGene00020771 | T24E12.1 | T24E12.1 | T24E12.1 |
| 53 | AB | WBGene00021316 | Y32H12A.8 | Y32H12A.8 | Y32H12A.8 |
| 54 | AB | WBGene00001979 | hmp-2 | K05C4.6 | hmp-2 |
| 55 | AB | WBGene00001493 | frm-7 | C51F7.1 | frm-7 |
| 56 | AB | WBGene00006805 | unc-73 | F55C7.7 | unc-73 |
| 57 | AB | WBGene00017760 | F23H11.4 | F23H11.4 | F23H11.4 |
| 58 | AB | WBGene00004855 | sma-1 | R31.1 | sma-1 |
| 59 | AB | WBGene00022697 | cyy-1 | ZK353.1 | ZK353.1 |
| 60 | AB | WBGene00001017 | dnc-1 | ZK593.5 | dnc-1 |
| 61 | AB | WBGene00013018 | Y48G10A.1 | Y48G10A.1 | Y48G10A.1 |
| 62 | AB | WBGene00001056 | dpf-3 | K02F2.1 | dpf-3 |
| 63 | AB | WBGene00013529 | Y73F8A.24 | Y73F8A.24 | Y73F8A.24 |
| 64 | AB | WBGene00015915 | C17G10.1 | C17G10.1 | C17G10.1 |
| 65 | AB | WBGene00000503 | cht-1 | C04F6.3 | cht-1 |
| 66 | AB | WBGene00000102 | akt-1 | C12D8.10 | akt-1 |
| 67 | AB | WBGene00011272 | R53.2 | R53.2 | R53.2 |
| 68 | AB | WBGene00016606 | exoc-7 | C43E11.8 | exoc-7 |
| 69 | AB | WBGene00010483 | K01G12.3 | K01G12.3 | K01G12.3 |
| 70 | AB | WBGene00016501 | C37C3.9 | C37C3.9 | C37C3.9 |
| 71 | AB | WBGene00015766 | C14C11.2 | C14C11.2 | C14C11.2 |
| 72 | AB | WBGene00011615 | lsd-1 | T08D10.2 | lsd-1 |
| 73 | AB | WBGene00003779 | nob-1 | Y75B8A.2 | nob-1 |
| 74 | AB | WBGene00002280 | let-2 | F01G12.5 | let-2 |
| 75 | AB | WBGene00015217 | tag-224 | B0496.8 | tag-224 |
| 76 | AB | WBGene00001651 | gon-2 | T01H8.5 | gon-2 |
| 77 | AB | WBGene00016868 | C52B9.8 | C52B9.8 | helicase |
| 78 | AB | WBGene00021853 | Y54F10AM.11 | Y54F10AM.11 | Y54F10AM.11 |
| 79 | AB | WBGene00013137 | Y53C10A.6 | Y53C10A.6 | Y53C10A.6 |
| 80 | AB | WBGene00000148 | aph-2 | ZC434.6 | aph-2 |

| **Rank** | **Cell** | **WBID** | **Public name** | **Sequence**  **name** | **RefSeq name** |
| --- | --- | --- | --- | --- | --- |
| 1 | P1 | WBGene00000496 | chs-1 | T25G3.2 | chs-1 |
| 2 | P1 | WBGene00021200 | cyp-31A3 | Y17G9B.3 | cyp-31A3 |
| 3 | P1 | WBGene00017671 | pgal-1 | F21F3.1 | F21F3.1 |
| 4 | P1 | WBGene00000550 | clu-1 | F55H2.6 | clu-1 |
| 5 | P1 | WBGene00019811 | egg-2 | R01H2.3 | egg-2 |
| 6 | P1 | WBGene00008914 | F17C11.2 | F17C11.2 | F17C11.2 |
| 7 | P1 | WBGene00010354 | cyp-31A2 | H02I12.8 | cyp-31A2 |
| 8 | P1 | WBGene00005022 | sqv-4 | F29F11.1 | sqv-4 |
| 9 | P1 | WBGene00004374 | rme-2 | T11F8.3 | rme-2 |
| 10 | P1 | WBGene00011350 | T01H3.4 | T01H3.4 | T01H3.4 |
| 11 | P1 | WBGene00020696 | T22F3.3 | T22F3.3 | T22F3.3 |
| 12 | P1 | WBGene00010036 | cpar-1 | F54C8.2 | cpar-1 |
| 13 | P1 | WBGene00020854 | T27C4.1 | T27C4.1 | T27C4.1 |
| 14 | P1 | WBGene00009264 | sac-1 | F30A10.6 | F30A10.6 |
| 15 | P1 | WBGene00001402 | fbf-2 | F21H12.5 | fbf-2 |
| 16 | P1 | WBGene00011225 | R10H10.7 | R10H10.7 | R10H10.7 |
| 17 | P1 | WBGene00010678 | K08F4.3 | K08F4.3 | K08F4.3 |
| 18 | P1 | WBGene00011409 | T04A8.7 | T04A8.7 | T04A8.7 |
| 19 | P1 | WBGene00017397 | F12A10.8 | F12A10.8 | F12A10.8 |
| 20 | P1 | WBGene00021486 | lbp-9 | Y40B10A.1 | lbp-9 |
| 21 | P1 | WBGene00013284 | daf-22 | Y57A10C.6 | Y57A10C.6 |
| 22 | P1 | WBGene00006984 | zig-7 | F54D7.4 | zig-7 |
| 23 | P1 | WBGene00006562 | tdc-1 | K01C8.3 | tdc-1 |
| 24 | P1 | WBGene00013765 | Y113G7B.16 | Y113G7B.16 | Y113G7B.16 |
| 25 | P1 | WBGene00019001 | F57B10.3 | F57B10.3 | F57B10.3 |
| 26 | P1 | WBGene00005026 | NA | NA | sqv-8 |
| 27 | P1 | WBGene00004268 | rab-5 | F26H9.6 | rab-5 |
| 28 | P1 | WBGene00012000 | T24H10.1 | T24H10.1 | T24H10.1 |
| 29 | P1 | WBGene00012031 | T25G3.4 | T25G3.4 | T25G3.4 |
| 30 | P1 | WBGene00015102 | cpg-2 | B0280.5 | cpg-2 |
| 31 | P1 | WBGene00019160 | H05L03.3 | H05L03.3 | H05L03.3 |
| 32 | P1 | WBGene00003994 | pgl-3 | C18G1.4 | pgl-3 |
| 33 | P1 | WBGene00015463 | C05C8.6 | C05C8.6 | C05C8.6 |
| 34 | P1 | WBGene00020721 | T23B12.6 | T23B12.6 | T23B12.6 |
| 35 | P1 | WBGene00015515 | spdl-1 | C06A8.5 | spdl-1 |
| 36 | P1 | WBGene00011392 | sbt-1 | T03D8.3 | sbt-1 |
| 37 | P1 | WBGene00019890 | R05F9.6 | R05F9.6 | phosphoglucomutase |
| 38 | P1 | WBGene00016262 | C30F12.3 | C30F12.3 | C30F12.3 |
| 39 | P1 | WBGene00014294 | C53C7.5 | C53C7.5 |  |
| 40 | P1 | WBGene00011986 | T24D1.3 | T24D1.3 | T24D1.3 |
| 41 | P1 | WBGene00010061 | F54E12.2 | F54E12.2 | helicase |
| 42 | P1 | WBGene00008990 | smgl-1 | F20G4.1 | smgl-1 |
| 43 | P1 | WBGene00006794 | unc-60 | C38C3.5 | unc-60 |
| 44 | P1 | WBGene00004266 | rab-1 | C39F7.4 | rab-1 |
| 45 | P1 | WBGene00004258 | pyc-1 | D2023.2 | pyc-1 |
| 46 | P1 | WBGene00001455 | flp-12 | C05E11.8 | flp-12 |
| 47 | P1 | WBGene00003750 | nlp-12 | M01D7.5 | nlp-12 |
| 48 | P1 | WBGene00011063 | cpg-3 | R06C7.4 | cpg-3 |
| 49 | P1 | WBGene00000585 | cogc-2 | C06G3.10 | cogc-2 |
| 50 | P1 | WBGene00019488 | K07D4.9 | K07D4.9 | K07D4.9 |
| 51 | P1 | WBGene00021093 | W08F4.3 | W08F4.3 | W08F4.3 |
| 52 | P1 | WBGene00004373 | rme-1 | W06H8.1 | rme-1 |
| 53 | P1 | WBGene00012716 | Y39E4B.5 | Y39E4B.5 | Y39E4B.5 |
| 54 | P1 | WBGene00000277 | cab-1 | C23H4.1 | cab-1 |
| 55 | P1 | WBGene00009476 | F36F2.2 | F36F2.2 | F36F2.2 |
| 56 | P1 | WBGene00001189 | egl-21 | F01D4.4 | egl-21 |
| 57 | P1 | WBGene00044638 | F23A7.8 | F23A7.8 | F23A7.8 |
| 58 | P1 | WBGene00005019 | sqv-1 | D2096.4 | sqv-1 |
| 59 | P1 | WBGene00010048 | F54D5.2 | F54D5.2 | F54D5.2 |
| 60 | P1 | WBGene00015083 | egg-1 | B0244.8 | egg-1 |
| 61 | P1 | WBGene00011383 | T02E9.5 | T02E9.5 | T02E9.5 |
| 62 | P1 | WBGene00018226 | F40B5.2 | F40B5.2 | F40B5.2 |
| 63 | P1 | WBGene00006419 | oac-39 | R02C2.3 | oac-39 |
| 64 | P1 | WBGene00009035 | F22B3.4 | F22B3.4 | F22B3.4 |
| 65 | P1 | WBGene00008887 | F16D3.4 | F16D3.4 | F16D3.4 |
| 66 | P1 | WBGene00000991 | dhs-28 | M03A8.1 | dhs-28 |
| 67 | P1 | WBGene00010424 | H36L18.2 | H36L18.2 | H36L18.2 |
| 68 | P1 | WBGene00003758 | nlp-20 | F45E4.8 | nlp-20 |
| 69 | P1 | WBGene00007130 | B0272.4 | B0272.4 | B0272.4 |
| 70 | P1 | WBGene00011543 | acl-2 | T06E8.1 | acl-2 |
| 71 | P1 | WBGene00021051 | W05H9.4 | W05H9.4 | W05H9.4 |
| 72 | P1 | WBGene00011352 | rskn-1 | T01H8.1 | rskn-1 |
| 73 | P1 | WBGene00013540 | Y75B8A.3 | Y75B8A.3 | Carboxylesterases |
| 74 | P1 | WBGene00011775 | T14G10.5 | T14G10.5 | T14G10.5 |
| 75 | P1 | WBGene00004239 | puf-3 | Y45F10A.2 | puf-3 |
| 76 | P1 | WBGene00011739 | T12G3.4 | T12G3.4 | T12G3.4 |
| 77 | P1 | WBGene00020230 | nep-2 | T05A8.4 | nep-2 |
| 78 | P1 | WBGene00019537 | K08D12.3 | K08D12.3 | K08D12.3 |
| 79 | P1 | WBGene00022497 | Y119D3B.21 | Y119D3B.21 | Y119D3B.21 |
| 80 | P1 | WBGene00000537 | clk-2 | C07H6.6 | clk-2 |
| 81 | P1 | WBGene00007228 | acs-7 | C01G6.7 | C01G6.7 |
| 82 | P1 | WBGene00016201 | C28H8.11 | C28H8.11 | C28H8.11 |
| 83 | P1 | WBGene00012306 | W06F12.2 | W06F12.2 | W06F12.2 |
| 84 | P1 | WBGene00007720 | C25D7.10 | C25D7.10 | C25D7.10 |
| 85 | P1 | WBGene00019505 | K07H8.3 | K07H8.3 | K07H8.3 |
| 86 | P1 | WBGene00008041 | C40H1.6 | C40H1.6 | C40H1.6 |
| 87 | P1 | WBGene00017283 | F09E5.3 | F09E5.3 | F09E5.3 |
| 88 | P1 | WBGene00011735 | hip-1 | T12D8.8 | T12D8.8 |
| 89 | P1 | WBGene00021286 | Y24D9A.8 | Y24D9A.8 | Y24D9A.8 |
| 90 | P1 | WBGene00000983 | dhs-20 | F35B12.2 | F35B12.3 |
| 91 | P1 | WBGene00022127 | yop-1 | Y71F9B.3 | yop-1 |
| 92 | P1 | WBGene00009940 | F52F12.7 | F52F12.7 | F52F12.7 |
| 93 | P1 | WBGene00004094 | ppw-2 | Y110A7A.18 | ppw-2 |
| 94 | P1 | WBGene00001566 | acdh-13 | C28C12.9 | acdh-13 |
| 95 | P1 | WBGene00020662 | T21H3.1 | T21H3.1 | T21H3.1 |
| 96 | P1 | WBGene00006935 | vars-1 | ZC513.4 | vrs-1 |
| 97 | P1 | WBGene00020498 | T14B4.1 | T14B4.1 | T14B4.1 |
| 98 | P1 | WBGene00010307 | F59B2.2 | F59B2.2 | F59B2.2 |
| 99 | P1 | WBGene00019017 | F57F4.4 | F57F4.4 | F57F4.4 |
| 100 | P1 | WBGene00018491 | mdh-1 | F46E10.10 | F46E10.10 |
| 101 | P1 | WBGene00000981 | dhs-18 | C45B11.3 | dhs-18 |
| 102 | P1 | WBGene00006574 | tin-13 | DY3.1 | tin-13 |
| 103 | P1 | WBGene00003759 | nlp-21 | Y47D3B.2 | nlp-21 |
| 104 | P1 | WBGene00021440 | Y39A3CL.1 | Y39A3CL.1 | Y39A3CL.1 |
| 105 | P1 | WBGene00010194 | btb-19 | F57C2.2 | btb-19 |
| 106 | P1 | WBGene00020910 | W01A11.2 | W01A11.2 | W01A11.2 |
| 107 | P1 | WBGene00004473 | rps-4 | Y43B11AR.4 | rps-4 |
| 108 | P1 | WBGene00018152 | acs-4 | F37C12.7 | acs-16 |
| 109 | P1 | WBGene00015565 | C07D8.6 | C07D8.6 | C07D8.6 |
| 110 | P1 | WBGene00007145 | B0334.5 | B0334.5 | B0334.5 |
| 111 | P1 | WBGene00022678 | sar-1 | ZK180.4 | sar-1 |
| 112 | P1 | WBGene00004061 | pmp-4 | T02D1.5 | pmp-4 |
| 113 | P1 | WBGene00010781 | NA | NA | K11H3.4 |
| 114 | P1 | WBGene00017970 | F32A5.4 | F32A5.4 | F32A5.4 |
| 115 | P1 | WBGene00004445 | rpl-31 | W09C5.6 | rpl-31 |
| 116 | P1 | WBGene00019892 | R05F9.9 | R05F9.9 | R05F9.9 |
| 117 | P1 | WBGene00019710 | M01E11.1 | M01E11.1 | M01E11.1 |
| 118 | P1 | WBGene00017814 | F26A10.2 | F26A10.2 | F26A10.2 |
| 119 | P1 | WBGene00004076 | pod-2 | W09B6.1 | pod-2 |
| 120 | P1 | WBGene00005020 | sqv-2 | Y110A2AL.14 | sqv-2 |
| 121 | P1 | WBGene00020549 | nmt-1 | T17E9.2 | nmt-1 |
| 122 | P1 | WBGene00004483 | rps-14 | F37C12.9 | rps-14 |
| 123 | P1 | WBGene00021035 | W05F2.3 | W05F2.3 | W05F2.3 |
| 124 | P1 | WBGene00008546 | F07A11.2 | F07A11.2 | F07A11.2 |
| 125 | P1 | WBGene00012585 | lips-15 | Y38E10A.7 | lips-15 |
| 126 | P1 | WBGene00019780 | M60.4 | M60.4 | M60.4 |
| 127 | P1 | WBGene00003898 | oxi-1 | Y39A1C.2 | oxi-1 |
| 128 | P1 | WBGene00022347 | Y82E9BR.14 | Y82E9BR.14 | Y82E9BR.14 |
| 129 | P1 | WBGene00004506 | rpt-6 | Y49E10.1 | rpt-6 |
| 130 | P1 | WBGene00000223 | atf-7 | C07G2.2 | atf-7 |
| 131 | P1 | WBGene00008118 | C46F11.3 | C46F11.3 | C46F11.3 |
| 132 | P1 | WBGene00004426 | rpl-14 | C04F12.4 | rpl-14 |
| 133 | P1 | WBGene00004485 | rps-16 | T01C3.6 | rps-16 |
| 134 | P1 | WBGene00004442 | rpl-28 | R11D1.8 | rpl-28 |
| 135 | P1 | WBGene00009559 | mtx-1 | F39B2.11 | mtx-1 |
| 136 | P1 | WBGene00022159 | mppa-1 | Y71G12B.24 | mppa-1 |
| 137 | P1 | WBGene00009119 | F25H2.5 | F25H2.5 | F25H2.5 |
| 138 | P1 | WBGene00021847 | Y54F10AL.1 | Y54F10AL.1 | Y54F10AL.1 |
| 139 | P1 | WBGene00001872 | him-14 | ZK1127.11 | him-14 |
| 140 | P1 | WBGene00003704 | nhr-114 | Y45G5AM.1 | nhr-114 |
| 141 | P1 | WBGene00017719 | F22F7.1 | F22F7.1 | F22F7.1 |
| 142 | P1 | WBGene00004272 | rab-8 | D1037.4 | rab-8 |
| 143 | P1 | WBGene00004198 | prx-13 | F32A5.6 | prx-13 |
| 144 | P1 | WBGene00019007 | F57B10.14 | F57B10.14 | F57B10.14 |
| 145 | P1 | WBGene00013143 | Y53C12B.1 | Y53C12B.1 | Y53C12B.1 |
| 146 | P1 | WBGene00021596 | spsb-2 | Y46E12BL.3 | Y46E12BL.3 |
| 147 | P1 | NA | NA | NA | Y54E2A.6 |
| 148 | P1 | WBGene00000777 | cpn-1 | F43G9.9 | cpn-1 |
| 149 | P1 | WBGene00012700 | Y39B6A.42 | Y39B6A.42 | Y39B6A.42 |
| 150 | P1 | WBGene00006916 | vha-7 | C26H9A.1 | vha-7 |
| 151 | P1 | WBGene00015716 | sox-4 | C12D12.5 | C12D12.5 |
| 152 | P1 | WBGene00004758 | sek-1 | R03G5.2 | sek-1 |
| 153 | P1 | WBGene00020447 | T12B3.4 | T12B3.4 | T12B3.4 |
| 154 | P1 | WBGene00004475 | rps-6 | Y71A12B.1 | rps-6 |
| 155 | P1 | WBGene00004492 | rps-23 | F28D1.7 | rps-23 |
| 156 | P1 | WBGene00004482 | rps-13 | C16A3.9 | rps-13 |
| 157 | P1 | WBGene00007087 | B0001.2 | B0001.2 | B0001.2 |
| 158 | P1 | WBGene00022075 | Y69A2AR.3 | Y69A2AR.3 | Y69A2AR.3 |
| 159 | P1 | WBGene00004421 | rpl-10 | F10B5.1 | rpl-10 |
| 160 | P1 | WBGene00008877 | F16A11.2 | F16A11.2 | F16A11.2 |
| 161 | P1 | WBGene00008169 | C48B4.6 | C48B4.6 | C48B4.6 |
| 162 | P1 | WBGene00016493 | C37A2.7 | C37A2.7 | C37A2.7 |
| 163 | P1 | WBGene00004924 | snt-4 | T23H2.2 | snt-4 |
| 164 | P1 | WBGene00001812 | haf-2 | F43E2.4 | haf-2 |
| 165 | P1 | WBGene00020601 | T20B12.3 | T20B12.3 | T20B12.3 |
| 166 | P1 | WBGene00022464 | Y110A7A.19 | Y110A7A.19 | Y110A7A.19 |
| 167 | P1 | WBGene00004409 | rla-1 | Y37E3.7 | rla-1 |
| 168 | P1 | WBGene00009888 | F49E2.5 | F49E2.5 | F49E2.5 |
| 169 | P1 | WBGene00021763 | Y51F10.2 | Y51F10.2 | Y51F10.2 |
| 170 | P1 | WBGene00000259 | bpl-1 | F13H8.10 | bpl-1 |
| 171 | P1 | WBGene00016987 | CC8.2 | CC8.2 | CC8.2 |
| 172 | P1 | WBGene00018349 | F42C5.9 | F42C5.9 | F42C5.9 |
| 173 | P1 | WBGene00008410 | D2023.6 | D2023.6 | D2023.6 |
| 174 | P1 | WBGene00017799 | F25G6.8 | F25G6.8 | F25G6.8 |
| 175 | P1 | WBGene00002180 | jtr-1 | Y77E11A.4 | jtr-1 |
| 176 | P1 | WBGene00004478 | rps-9 | F40F8.10 | rps-9 |
| 177 | P1 | WBGene00004044 | plk-3 | F55G1.8 | plk-3 |
| 178 | P1 | WBGene00021427 | Y38F2AR.9 | Y38F2AR.9 | Y38F2AR.9 |
| 179 | P1 | WBGene00006707 | ubc-12 | R09B3.4 | ubc-12 |
| 180 | P1 | WBGene00003370 | mlc-2 | C36E6.5 | mlc-2 |
| 181 | P1 | WBGene00008860 | rmo-1 | F15D4.3 | F15D4.3 |
| 182 | P1 | WBGene00004446 | rpl-32 | T24B8.1 | rpl-32 |
| 183 | P1 | WBGene00004477 | rps-8 | F42C5.8 | rps-8 |
| 184 | P1 | WBGene00002025 | hsp-60 | Y22D7AL.5 | hsp-60 |
| 185 | P1 | WBGene00021350 | Y37E3.8 | Y37E3.8 | Y37E3.8 |
| 186 | P1 | WBGene00004499 | rps-30 | C26F1.4 | rps-30 |
| 187 | P1 | WBGene00004491 | rps-22 | F53A3.3 | rps-22 |
| 188 | P1 | WBGene00000204 | arx-6 | C35D10.16 | arx-6 |
| 189 | P1 | WBGene00010579 | K05C4.2 | K05C4.2 | K05C4.2,K05C4.11 |
| 190 | P1 | WBGene00001746 | gsk-3 | Y18D10A.5 | gsk-3 |
| 191 | P1 | WBGene00022172 | Y71H2AM.7 | Y71H2AM.7 | Y71H2AM.7 |
| 192 | P1 | WBGene00021789 | nol-6 | Y51H7C.11 | nol-6 |
| 193 | P1 | WBGene00009562 | flp-22 | F39H2.1 | flp-22 |
| 194 | P1 | WBGene00016418 | C34G6.1 | C34G6.1 | C34G6.1 |
| 195 | P1 | WBGene00013882 | ZC410.5 | ZC410.5 | ZC410.5 |
| 196 | P1 | WBGene00008978 | cutl-3 | F20D1.8 | cutl-3 |
| 197 | P1 | WBGene00004481 | rps-12 | F54E7.2 | rps-12 |
| 198 | P1 | WBGene00015404 | C03H5.2 | C03H5.2 | C03H5.2 |
| 199 | P1 | WBGene00003989 | pfn-1 | Y18D10A.20 | pfn-1 |
| 200 | P1 | WBGene00016960 | vps-33.2 | C56C10.1 | vps-33.2 |
| 201 | P1 | WBGene00004085 | pph-4.1 | Y75B8A.30 | pph-4.1 |
