# Supplementary material for: Asymmetric Transcript Discovery by RNA-seq in C. elegans Blastomeres Identifies neg-1, a Gene Important for Anterior Morphogenesis
Source: PLoS Genet. 2015 Apr 13;11(4):e1005117. doi: 10.1371/journal.pgen.1005117 (PMC4395330; doi:10.1371/journal.pgen.1005117)
Supplement: S1 Text — A pdf document listing the rubric used to instruct reviewers how to score the transcripts were queried in the Kohara database and their patterns of in situ hybridization at the 2-cell stage of development. (PDF) [file pgen.1005117.s015.pdf]

The Categories

You will be able to select one of seven categories:

- AB
- marginal AB
- symmetric
- marginal P1
- P1
- no expression
- ambiguous

Assign a category to each gene using these criteria:

AB

clear AB bias in staining in two or more 2-cell stage embryos.

marginal AB

potentially some AB bias staining in more than one 2-cell stage embryo but also some ambiguity due to either faint differences in staining, too few 2-cell stage embryos, a lack in consistency, or difficulty in calling AB or P1 for a minority of embryos.

symmetric

even staining between AB and P1 cells. This can occur at different staining levels.

marginal P1

potentially some P1 bias staining in more than one 2-cell stage embryo but also some ambiguity due to either faint differences in staining, too few 2-cell stage embryos, a lack in consistency, or a difficulty in calling AB or P1 for a minority of embryos.

P1

clear P1 bias in staining in two or more 2-cell stage embryos.

no expression

no staining discernible at the 2-cell stage or staining too faint.

ambiguous

too many inconsistencies to categorize between. These inconsistencies could be due to patterning, staining, or image quality. This category includes a mix of situations including (1) instances where some embryos have AB-biased and others have P1-biased staining, or (2) some images are successfully stained and others are not or (3) embryos are too dark or too out of focus to score.
